# Supplementary material for: Multidimensional social influence drives leadership and composition-dependent success in octopus–fish hunting groups
Source: Nat Ecol Evol. 2024 Sep 23;8(11):2072–84. doi: 10.1038/s41559-024-02525-2 (PMC11541198; doi:10.1038/s41559-024-02525-2)
Supplement: Supplementary file 1 — Supplementary Figs. 1–19 and Tables 1–52. [file 41559_2024_2525_MOESM1_ESM.pdf]

nature ecology & evolution

Supplementary information https://doi.org/10.1038/s41559-024-02525-2

Multidimensional social influence drives leadership and composition-dependent success in octopus–fish hunting groups

In the format provided by the authors and unedited

Supplementary Materials

The PDF file includes:

Supplementary Figures 1-19
Supplementary Tables 1-52

Other Supplementary Materials for this manuscript include the following:

Supplementary Videos 1-7
Supplementary Data 1-5
Supplementary Code 1-7

Supplementary Figure 1. Punctuated movement. Individual and group centroid speed profiles across time (annotated at 3 frames per second) for one group hunting scene, showing stop-and-movement dynamics. Visualization performed using the same group shown in Video S2.

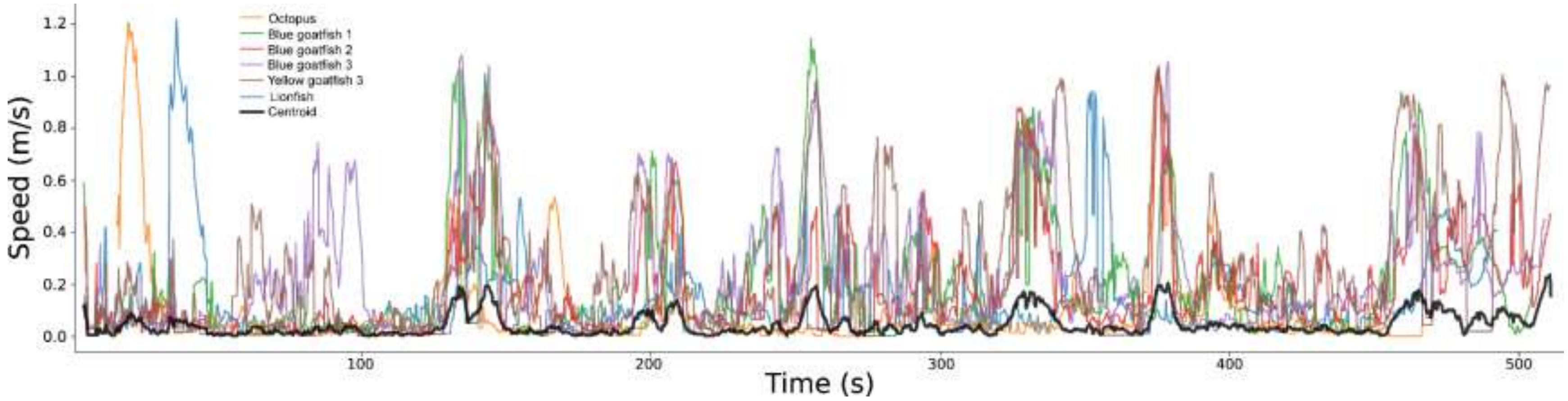

Supplementary Figure 2. Pull-Anchor outcomes. Species-specific performance in each subgroup regarding: (**A)** times pulling per minute (Supplementary Table 2), (**B)** times anchoring per minute (Supplementary Table 6), **(C)** times being pulled as a follower per minute (Supplementary Table 5), and **(D)** times anchored per minute (Supplementary Table 3). Values were also normalized considering the number of individuals that were present in each subgroup. Moveover, we also looked at pull-anchor ratios according to species, such as: (**E)** pulls divided by total number of initiations (Pulling efficiency) (Supplementary Table 7), **(F)** anchoring others divided by times where individuals could decide to follow or anchor (Supplementary Table 8), **(G)** pulling divided by following (Supplementary Table 9), and (**H)** initiations divided by following (Supplementary Table 10). Data points, violin plots (for ratios varying between 0 and 1), and boxplots are used. For boxplots, boxes represent the 25% and 75% quartiles, with the center (50%) being the median, and red dots indicating the mean. Whiskers represent the equal or lower/upper value of 1.5 * the interquartile range (between 25-75%). In the latter two panels, red dashed line in represent initiation (attempts)-followership threshold, where values >1 indicate a stronger leadership role and values <1 indicate a stronger role in the context of the group's movement initiation. Different letters indicate significant differences ($p < 0.05$).

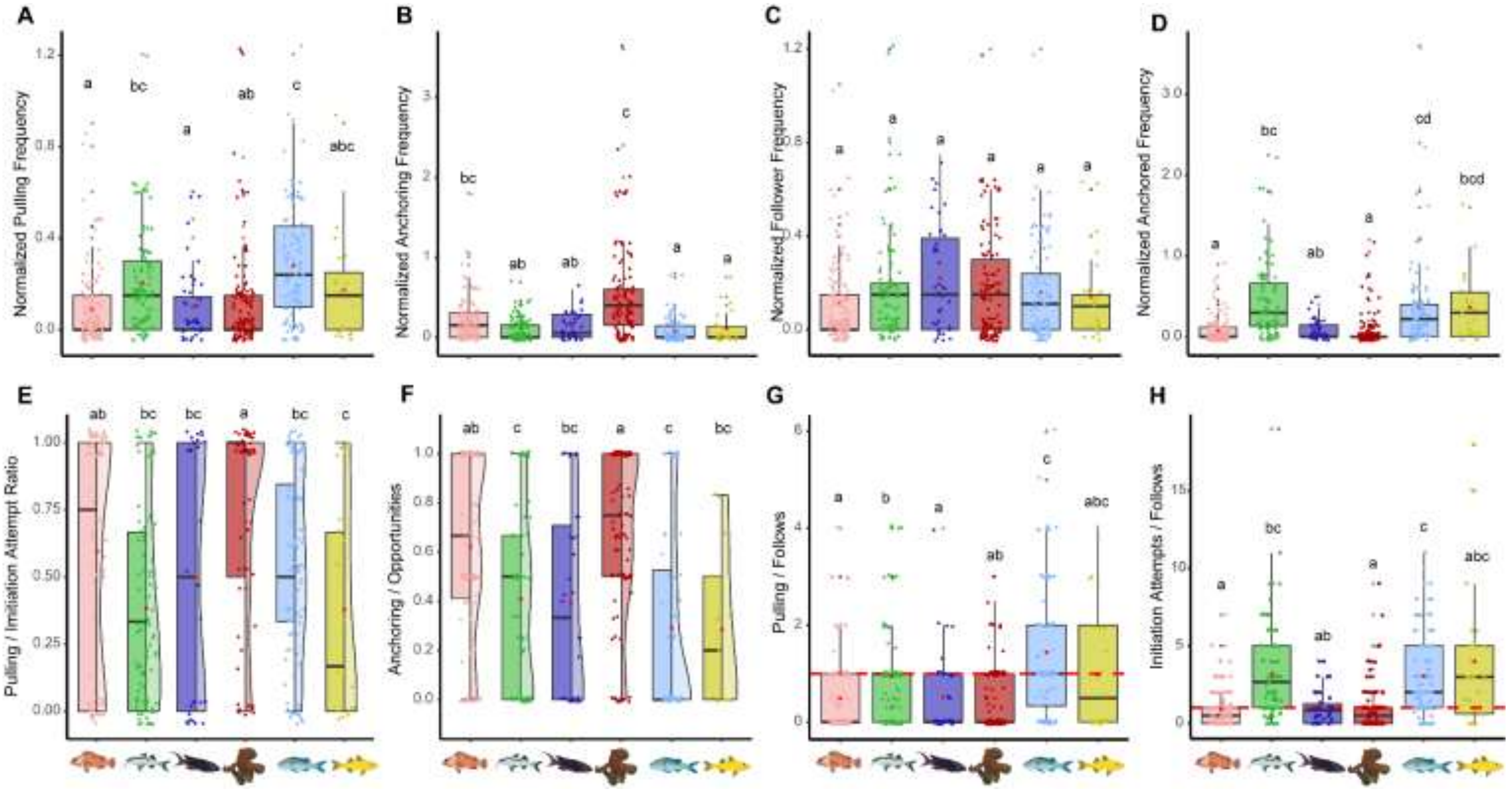
A
Normalized Pulling Frequency
B
Normalized Anchoring Frequency
C
Normalized Follower Frequency
D
Normalized Anchored Frequency
E
Pulling / Imitiation Attempt Ratio
F
Anchoring / Opportunities
G
Pulling / Follows
H
Initiation Attempts / Follows

Supplementary Figure 3. Frequency of 1st pulls. Boxplots and datapoints depicting species-specific times pulling per minute in each subgroup. In this case, for any given follower, pulling counts were limited to only the 1st puller, i.e. the first initiator that moved and was followed. Values were normalized considering the number of individuals that were present in each subgroup. Different letters indicate significant differences ($p < 0.05$, Supplementary Table 11). For boxplots, boxes represent the 25% and 75% quartiles, with the center (50%) being the median, and red dots indicating the mean. Whiskers represent the equal or lower/upper value of 1.5 * the interquartile range (between 25-75%).

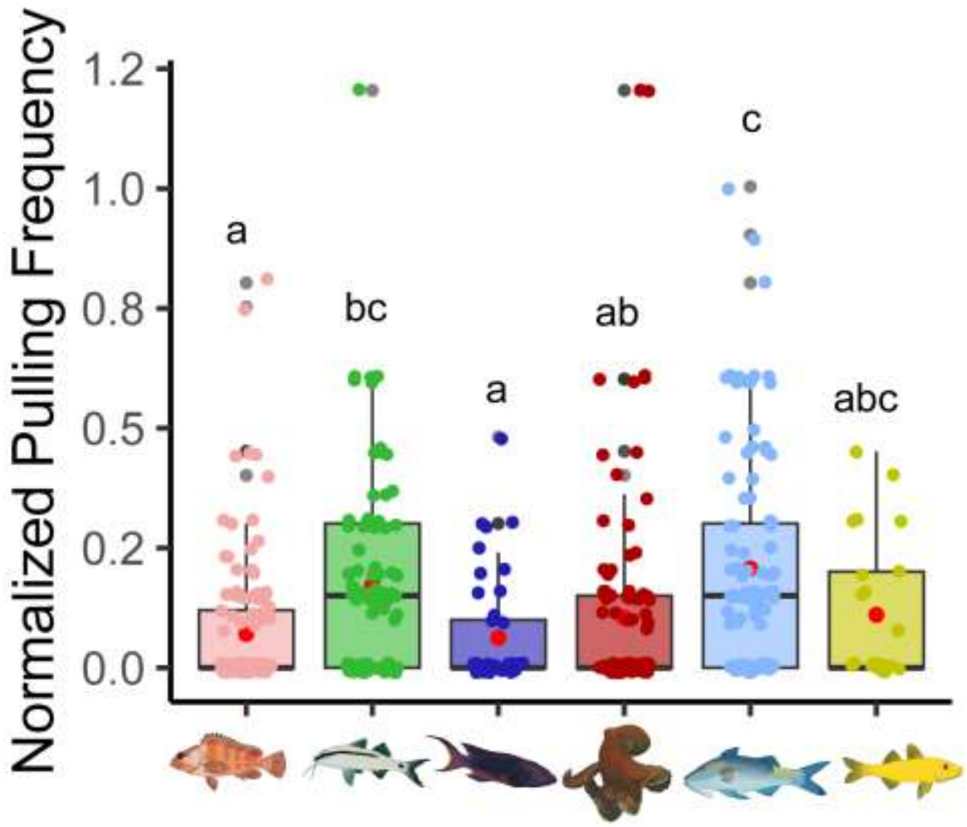

Supplementary Figure 4. Frequency of interspecific pulls. Boxplots and datapoints depicting species-specific times pulling per minute in each subgroup. In this case, pulling counts were limited to interspecific interactions, and same species interactions were removed. Values were normalized considering the number of individuals that were present in each subgroup. Different letters indicate significant differences ($p < 0.05$, Supplementary Table 12). For boxplots, boxes represent the 25% and 75% quartiles, with the center (50%) being the median, and red dots indicating the mean. Whiskers represent the equal or lower/upper value of 1.5 * the interquartile range (between 25-75%).

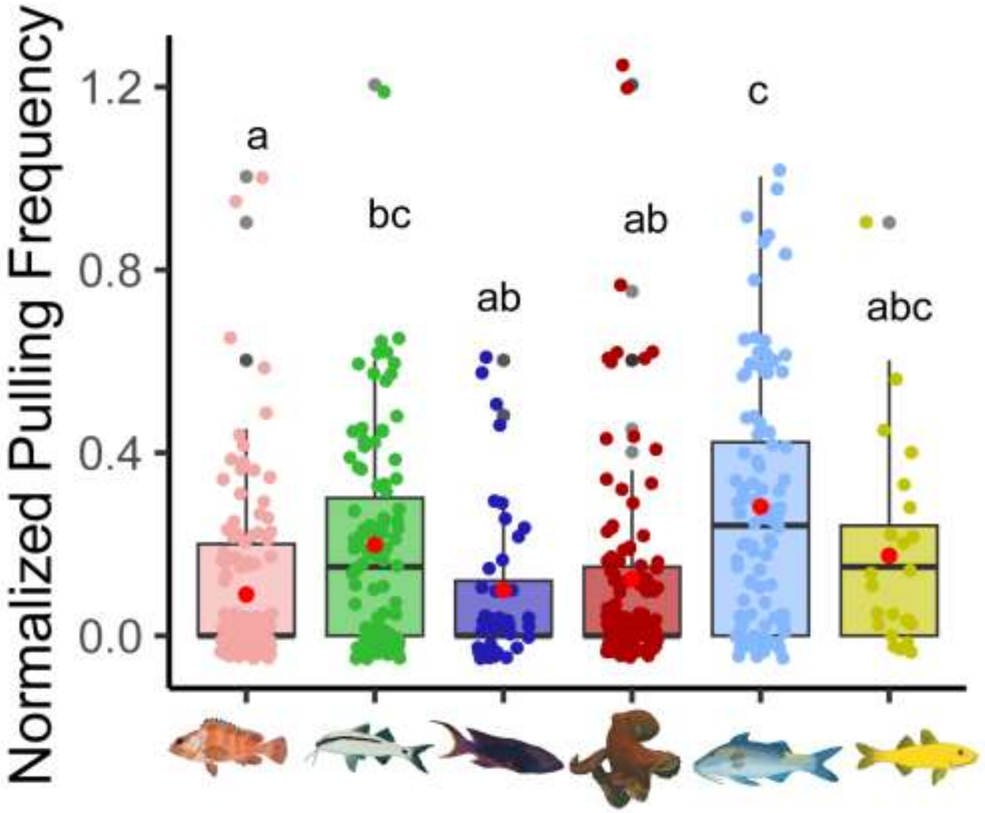

Supplementary Figure 5. Proportion of time spent immobile. Average amount of time spent immobile (considered as speeds below 1 cm s^{-1}) from the total amount of time present in a multispecific hunting subgroup. Letters show significant differences (Supplementary Table 13). Data points, violin plots, and boxplots are used. For boxplots, boxes represent the 25% and 75% quartiles, with the center (50%) being the median, and red dots indicating the mean. Whiskers represent the equal or lower/upper value of 1.5 * the interquartile range (between 25-75%).

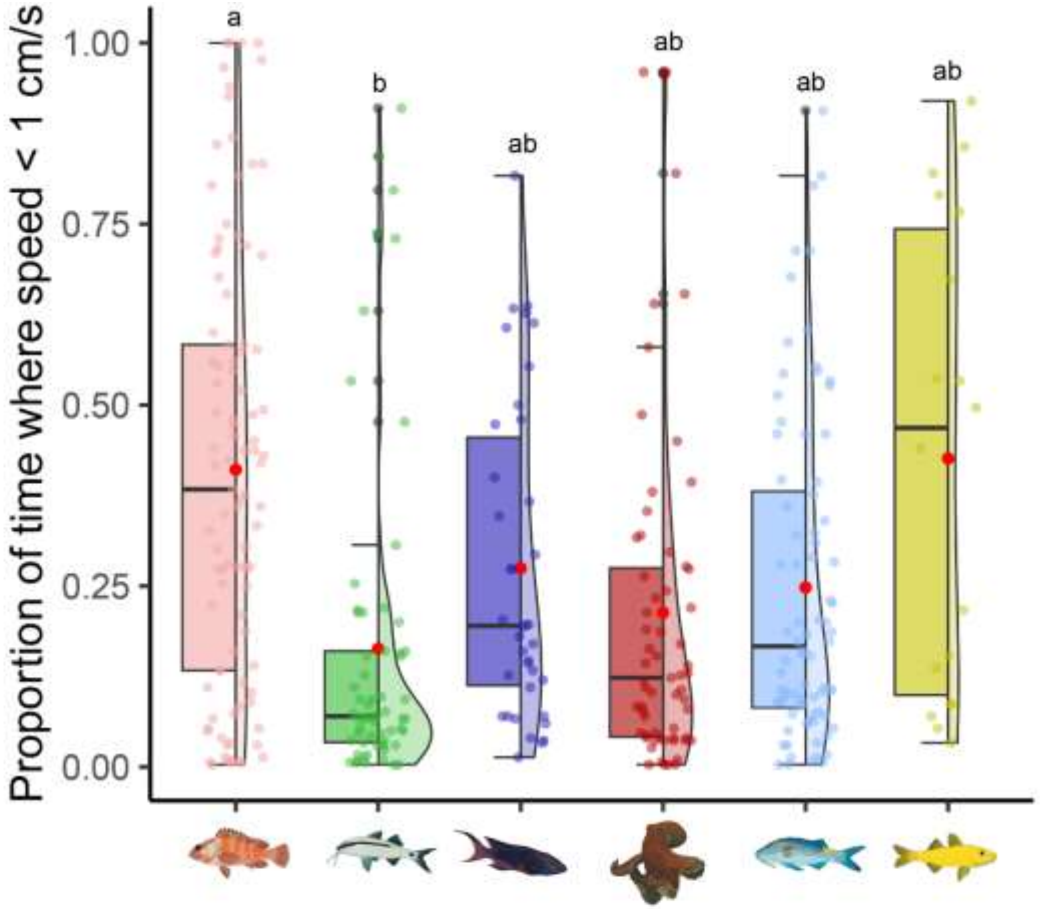

Supplementary Figure 6. Group properties in the presence and absence of blacktips. Boxplots and correlational scatterplots depicting significant differences between centroid displacement and mean distance to centroid in groups with (opaque icon, dark red) and without (transparent icon, light red) blacktips (Tables S19-20). Pearson's R and p-value are given in the figure. **** indicate significant differences p < 0.0001. Data points and boxplots are used. For boxplots, boxes represent the 25% and 75% quartiles, with the center (50%) being the median, and red dots indicating the mean. Whiskers represent the equal or lower/upper value of 1.5 * the interquartile range (between 25-75%).

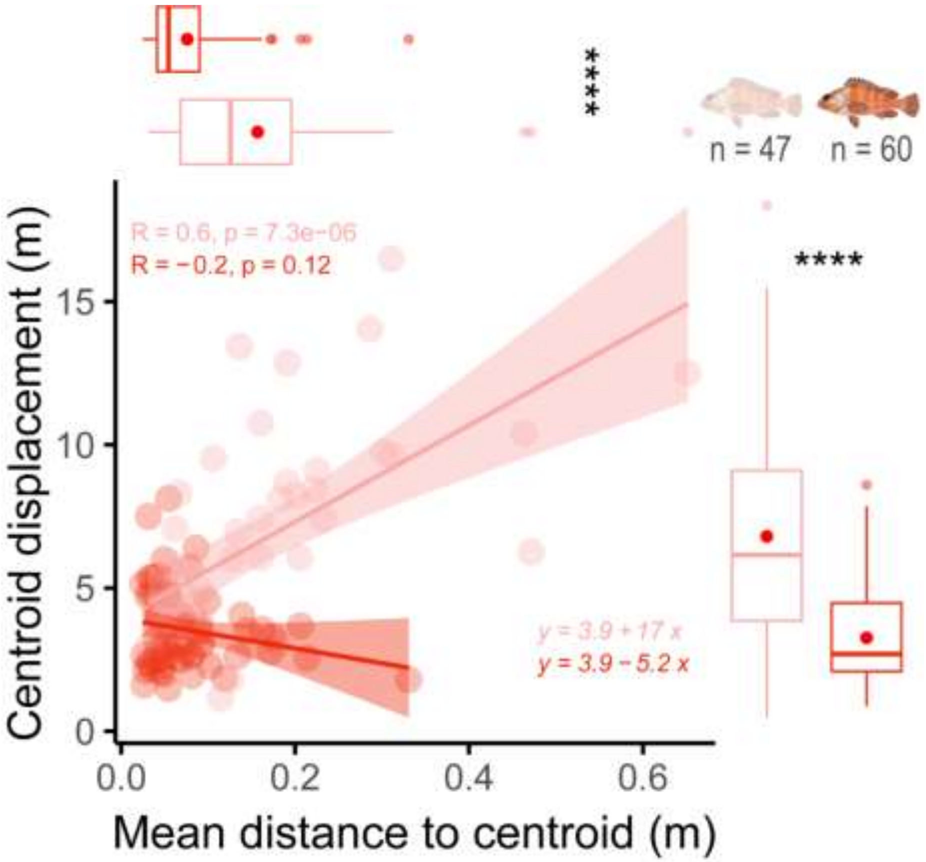

Supplementary Figure 7. Anchor frequency in the presence and absence of blue goatfish. Significant differences at individual-level in the frequency of anchors (normalized by the number of individuals present) in groups with (opaque icon, dark blue) and without (transparent icon, light blue) (Supplementary Table 22). **** indicate significant differences $p < 0.0001$. Data points, violin plots, and boxplots are used. For boxplots, boxes represent the 25% and 75% quartiles, with the center (50%) being the median, and red dots indicating the mean. Whiskers represent the equal or lower/upper value of 1.5 * the interquartile range (between 25-75%).

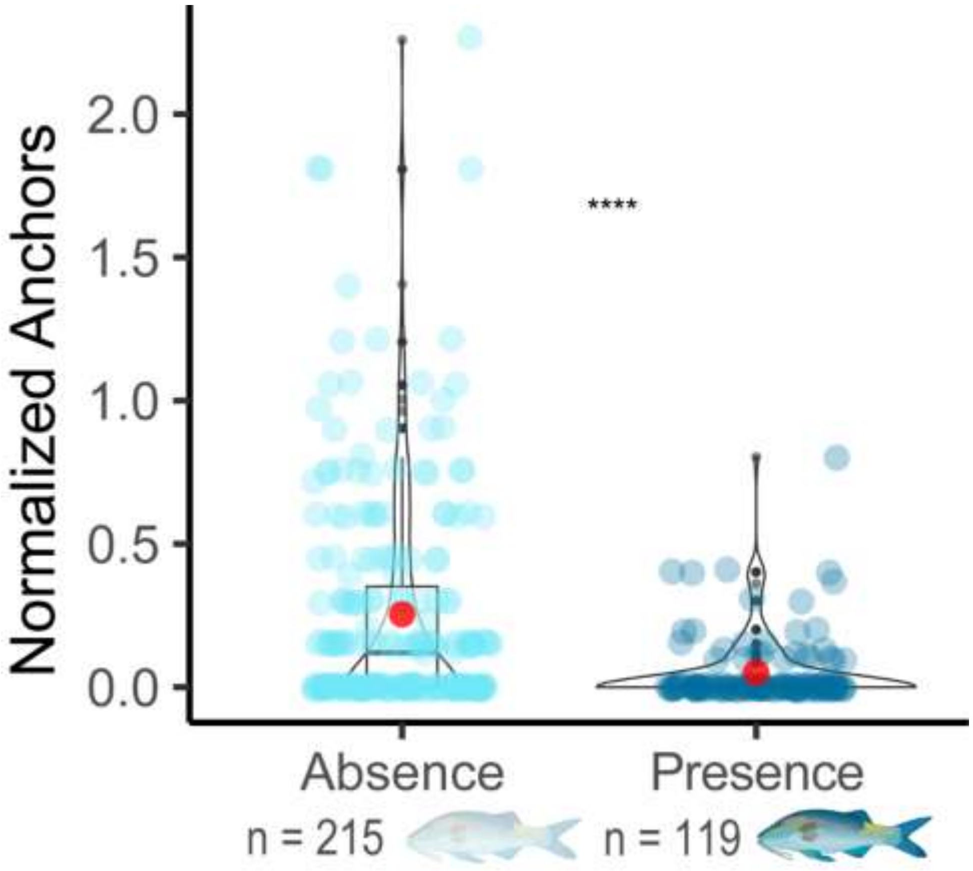

Supplementary Figure 8. Species-level pull-anchor results in the presence and absence of blue goatfish. Significant differences at species-level in the frequency (normalized by the number of individuals present) of initiations and anchors, as well as pulling efficiency, in groups with (opaque icon, dark blue) and without (transparent icon, light blue) blue goatfish (Tables S21-22). Data points, violin plots, and boxplots are used. For boxplots, boxes represent the 25% and 75% quartiles, with the center (50%) being the median, and red dots indicating the mean. Whiskers represent the equal or lower/upper value of 1.5 * the interquartile range (between 25-75%). Statistical significance shown as '****' for p < 0.0001, '***' for p < 0.001, '**' for p < 0.01, and '*' for p < 0.05.

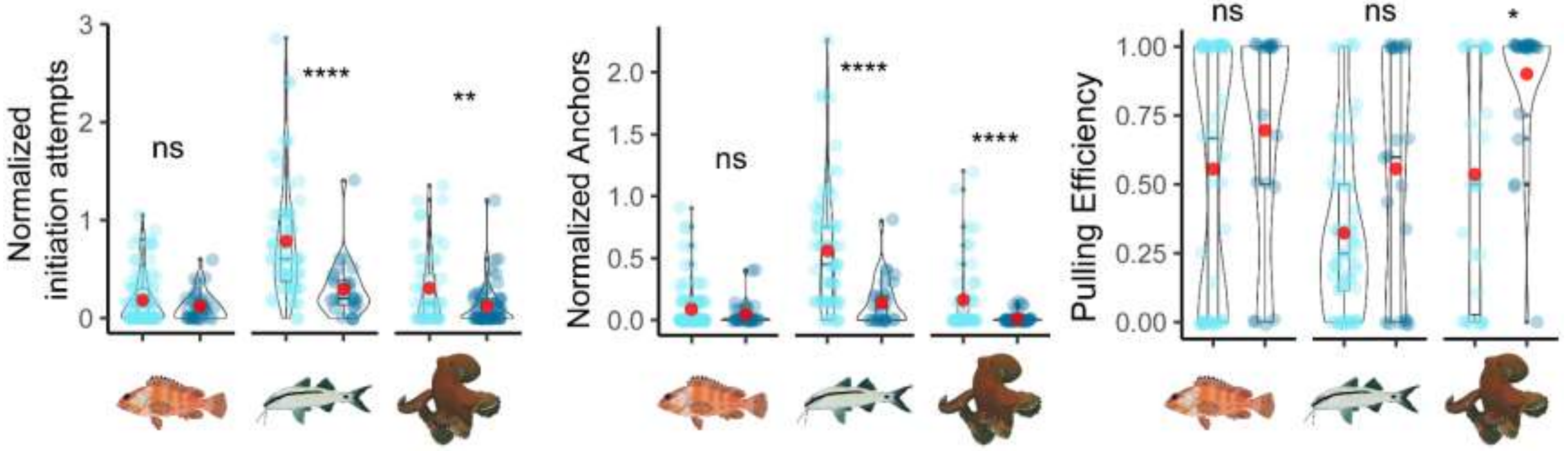

Supplementary Figure 9. Group properties in the presence and absence of blue goatfish. Boxplots and correlational scatterplots depicting significant differences between centroid displacement (Supplementary Table 24) and mean distance to centroid (Supplementary Table 25) in groups with (opaque icon, dark blue) and without (transparent icon, light blue) blue goatfish. Pearson's R and p-value are given in the figure. **** indicate significant differences p < 0.0001. Data points and boxplots are used. For boxplots, boxes represent the 25% and 75% quartiles, with the center (50%) being the median, and red dots indicating the mean. Whiskers represent the equal or lower/upper value of 1.5 * the interquartile range (between 25-75%).

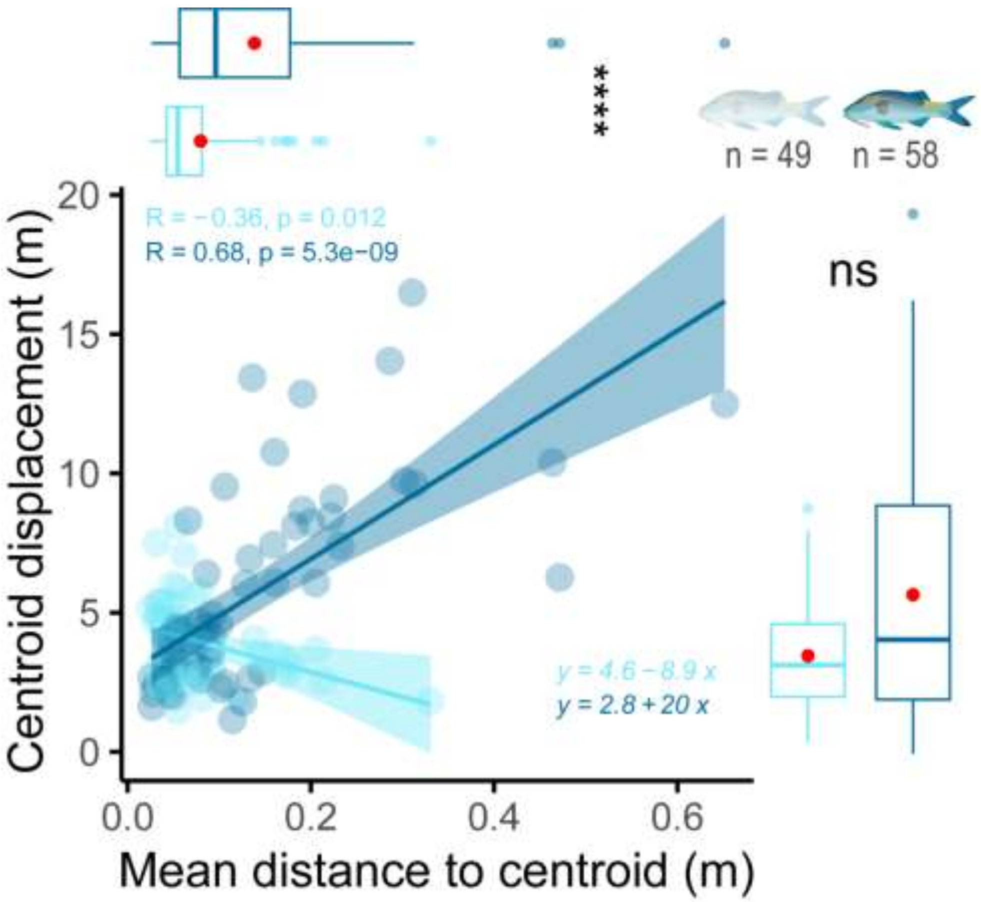

Supplementary Figure 10. Kinematics and characteristics of pulling. Differences in **(A)** speed, **(B)** distance to centroid **(C)** tortuosity, and **(D)** angle to centroid, according to the outcome of the initiation: anchor (unsuccessful) or pull (successful) (Tables S28). Data points, violin plots, and boxplots are used. For boxplots, boxes represent the 25% and 75% quartiles, with the center (50%) being the median, and red dots indicating the mean. Whiskers represent the equal or lower/upper value of 1.5 * the interquartile range (between 25-75%). Statistical significance shown as '****' for p < 0.0001, '***' for p < 0.001, '**' for p < 0.01, and '*' for p < 0.05.

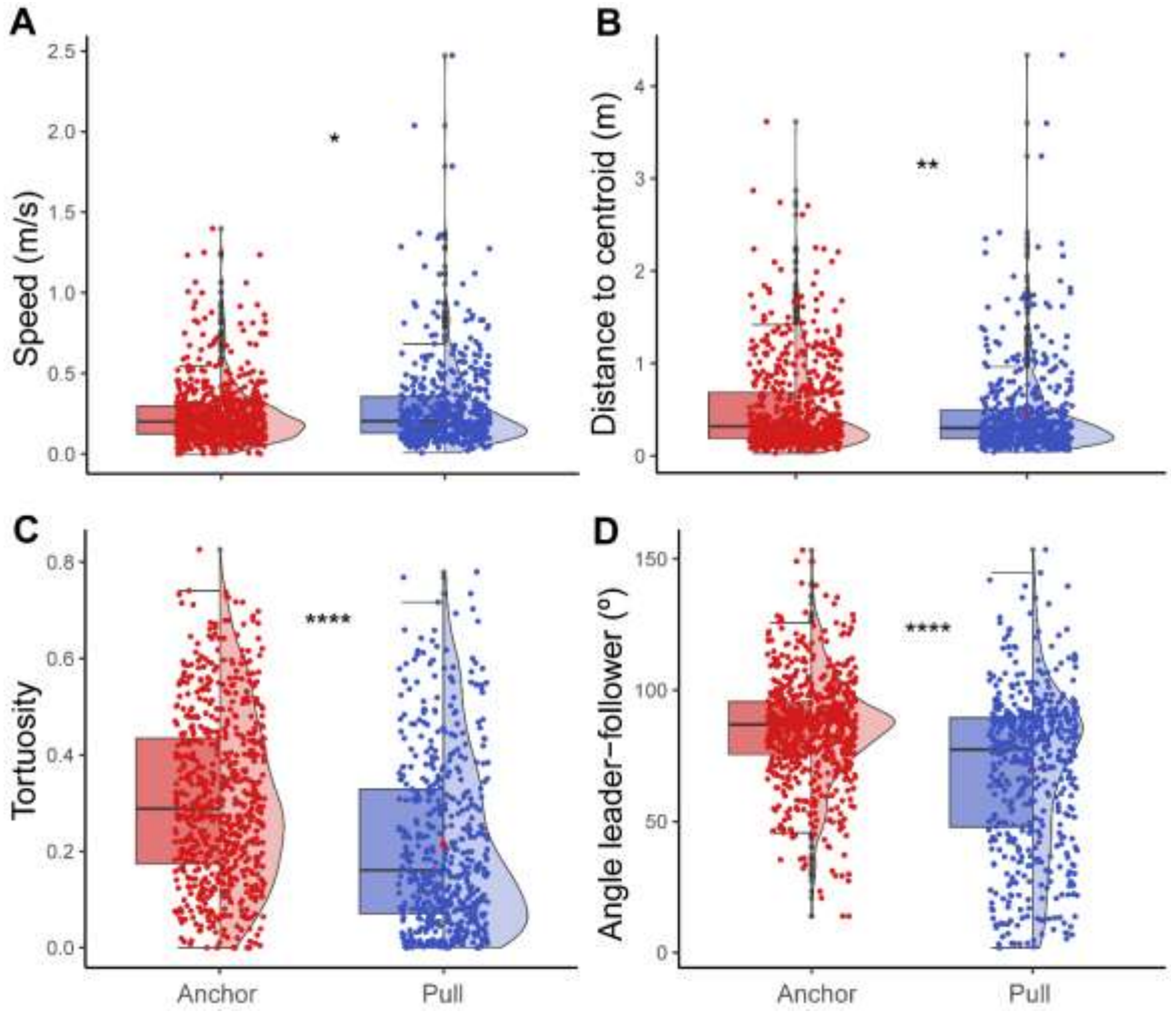

Supplementary Figure 11. Individual-level characteristics across hunting groups. (A) speed, **(B)** distance to centroid **(C)** tortuosity, and **(D)** angle to centroid, according to species during the overall time present in the hunting subgroup. Letters show significant differences (Tables S29-S32). Data points, violin plots, and boxplots are used. For boxplots, boxes represent the 25% and 75% quartiles, with the center (50%) being the median, and red dots indicating the mean. Whiskers represent the equal or lower/upper value of 1.5 * the interquartile range (between 25-75%).

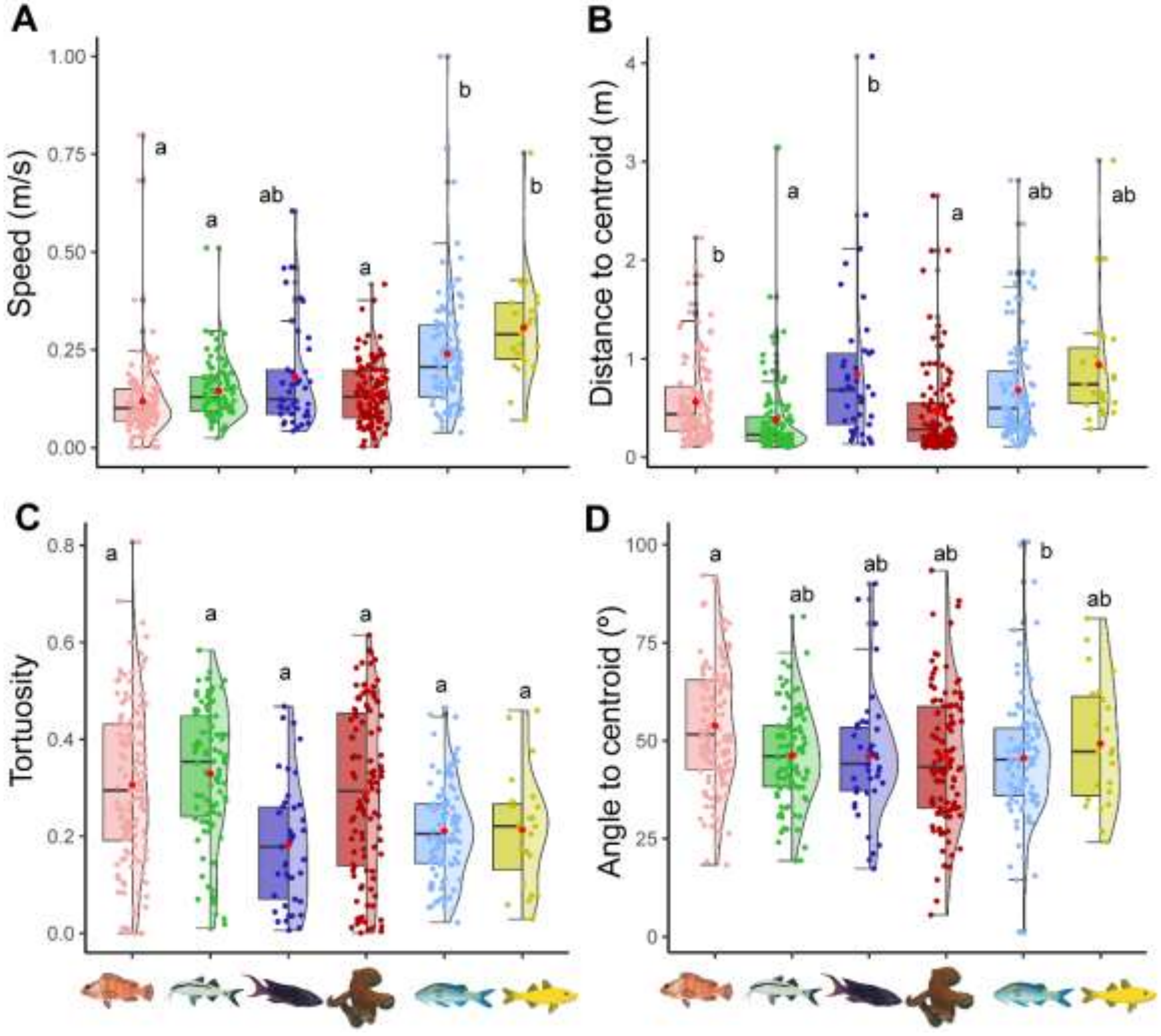

Supplementary Figure 12. Individual-level characteristics during initiations. Boxplots and violin plots depicting **(A)** speed, **(B)** distance to centroid **(C)** tortuosity, and **(D)** angle to centroid, according to species while performing an initiation (between $t1$ and $t2$). Letters show significant differences (Tables S33-S36). Data points, violin plots, and boxplots are used. For boxplots, boxes represent the 25% and 75% quartiles, with the center (50%) being the median, and red dots indicating the mean. Whiskers represent the equal or lower/upper value of 1.5 * the interquartile range (between 25-75%).

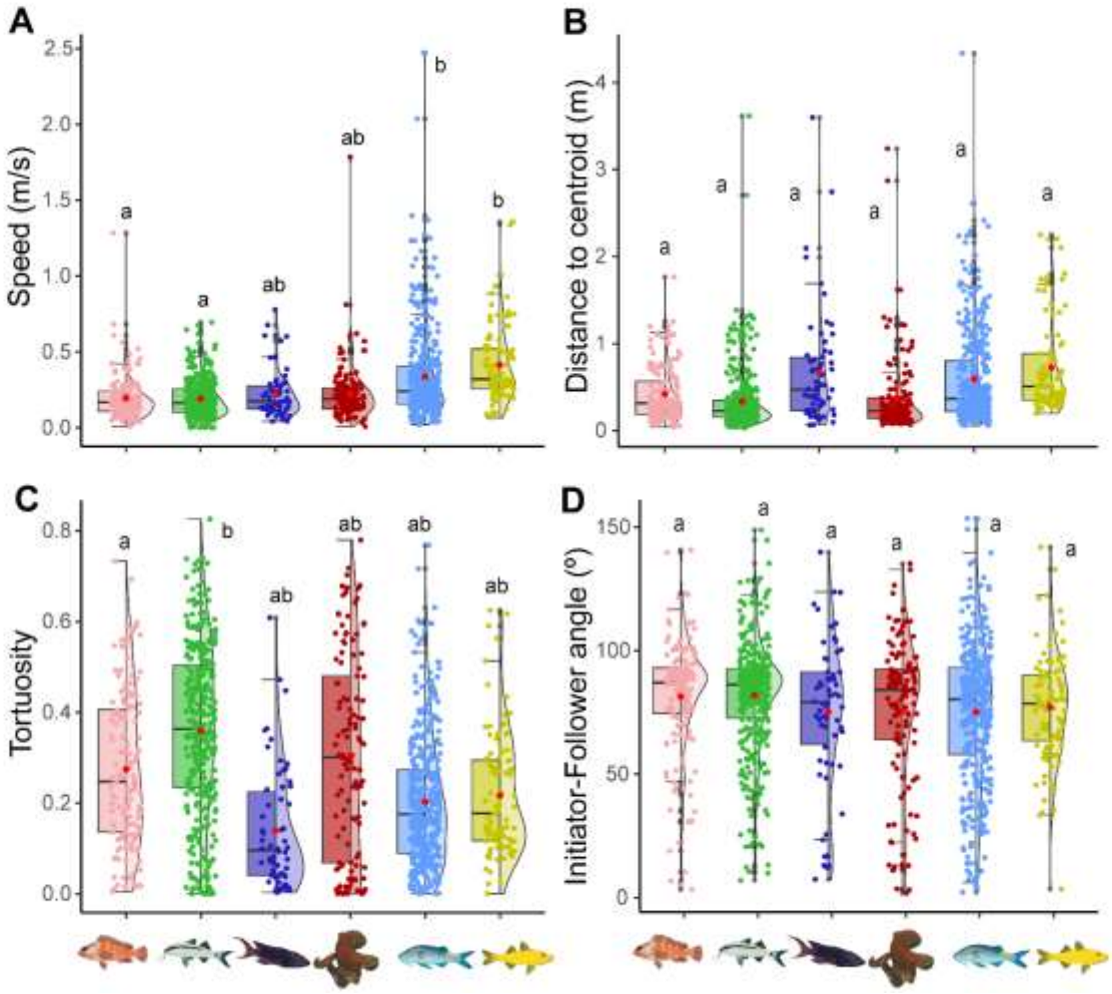

Supplementary Figure 13. Probability of punching occurring in groups with or without extreme phenotypes. Calculated probability of punching occurring in groups (binomial tests), depending on the presence/absence of **(A)** blacktips (Supplementary Table 39), and **(B)** blue goatfish (Supplementary Table 40). Statistical significance shown as '****' for $p < 0.0001$, '***' for $p < 0.001$, '**' for $p < 0.01$, and '*' for $p < 0.05$.

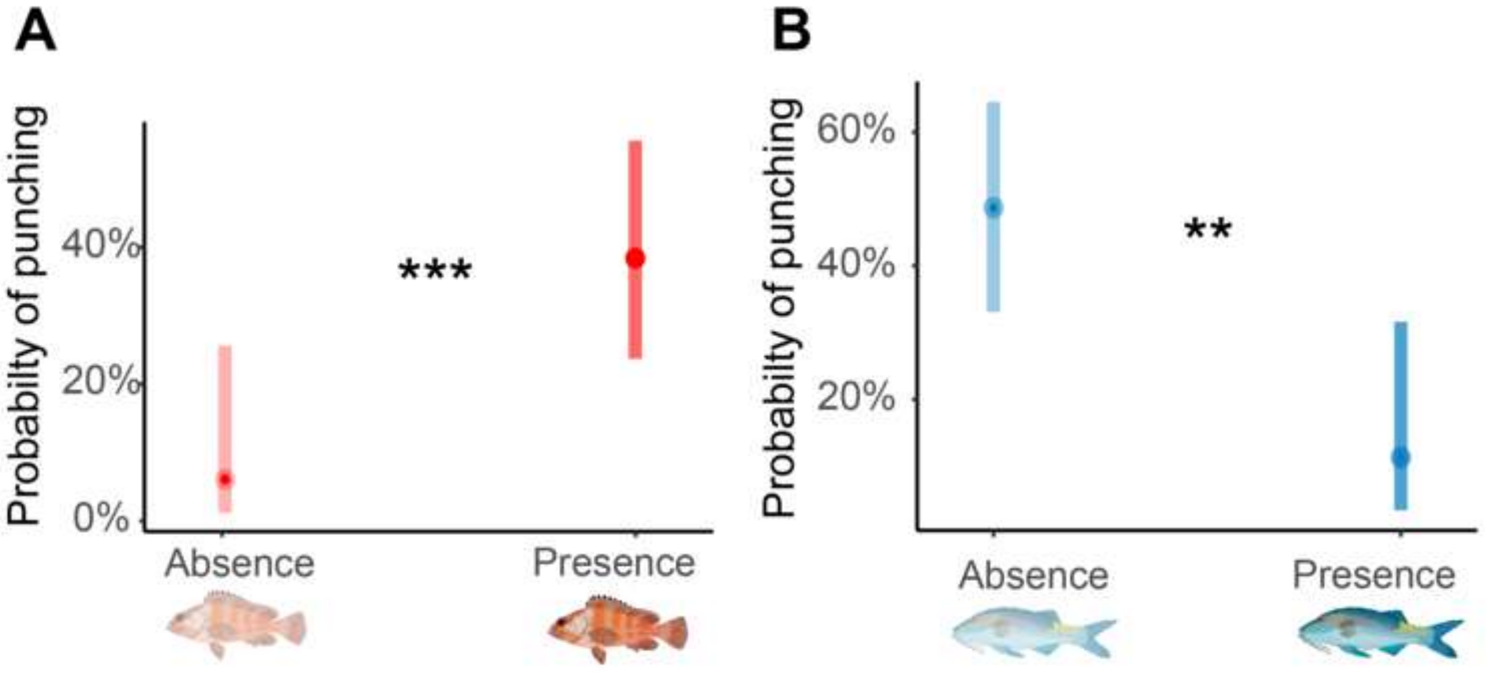

Supplementary Figure 14. Punching model for extreme phenotypes. Estimates, confidence intervals, and significant differences (red arrows) regarding the probability of punching ocurring, depending on the factorial combination of presence/absence of extreme phenotypes (Supplementary Table 41).

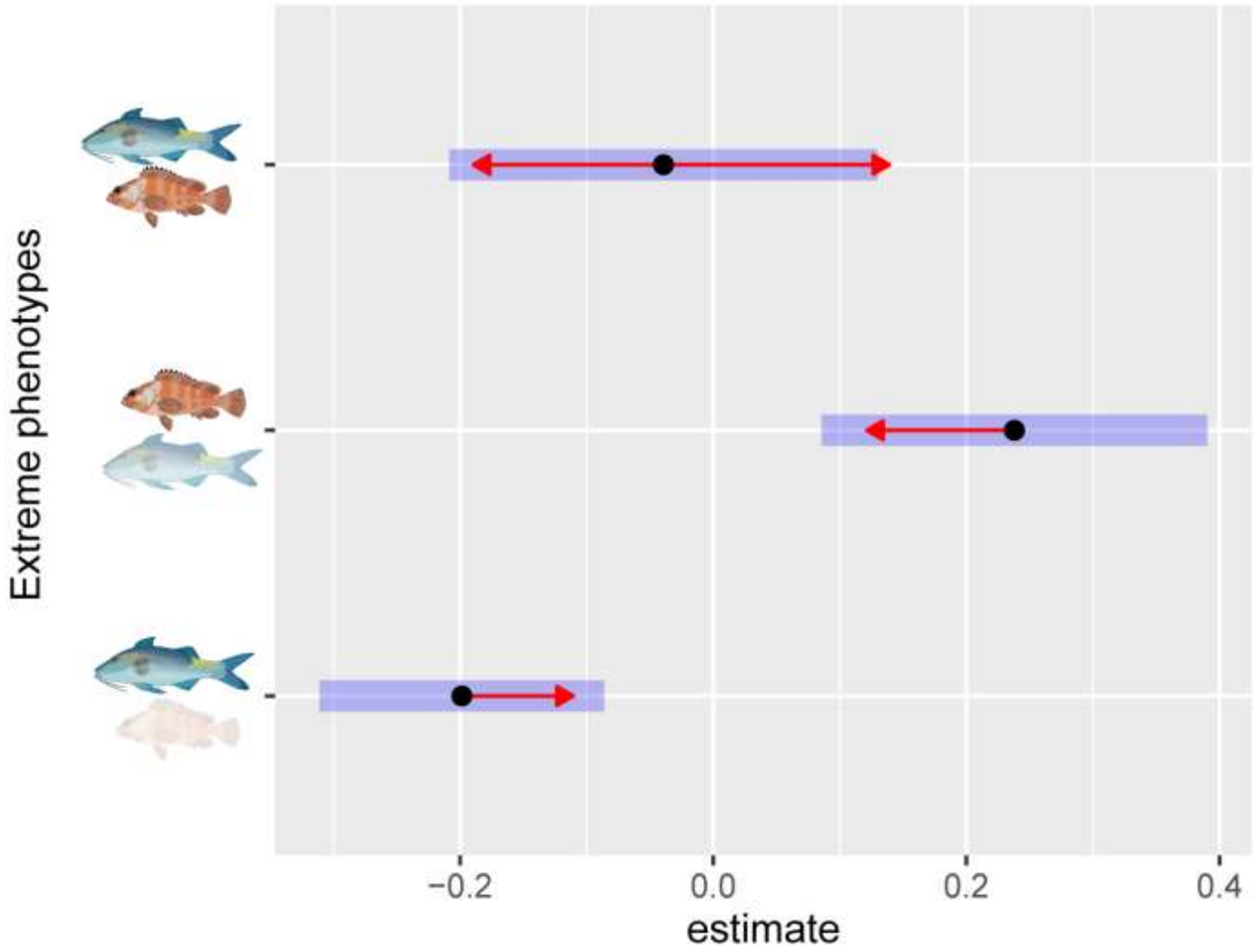

Supplementary Figure 15. Field experiment setup. U-PVC tee-fitting structures used to evaluate the temporal characteristics of octopus web-overs. The experimental design entailed two conditions: **(A)** empty, and **(B)** full (or food-baited) structures.

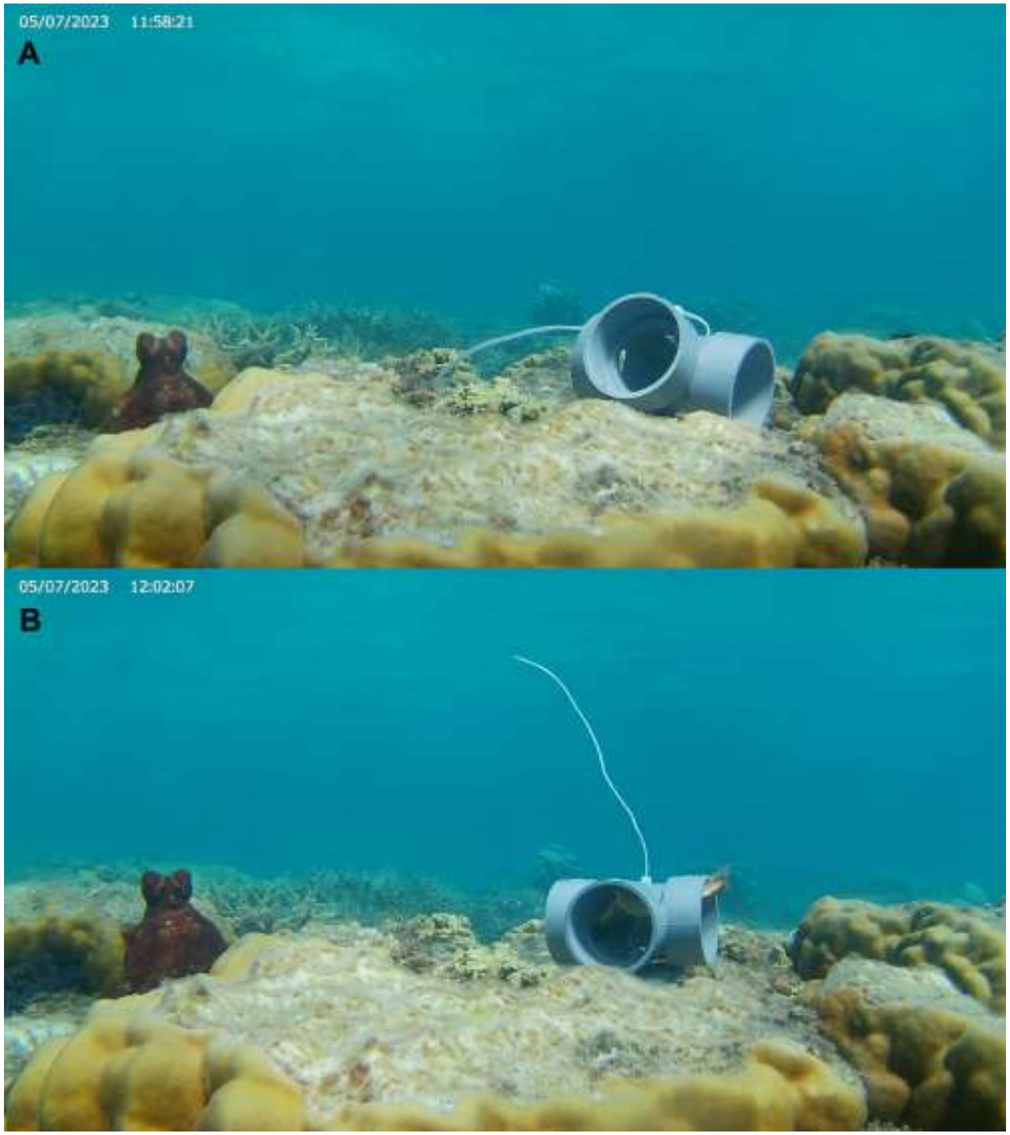

Supplementary Figure 16. Probability of octopuses performing web-overs does not depend on structures containing food, but on previous fish attack. Calculated predictions (binomial tests) based on experimental results demonstrating that decisions to perform web-overs on structures **(A)** did not depend on structure status (empty or full), **(B)** but on structures being previously attacked by fish (Supplementary Table 43). Statistical significance shown as '**' for $p < 0.01$ and 'ns' for $p > 0.05$.

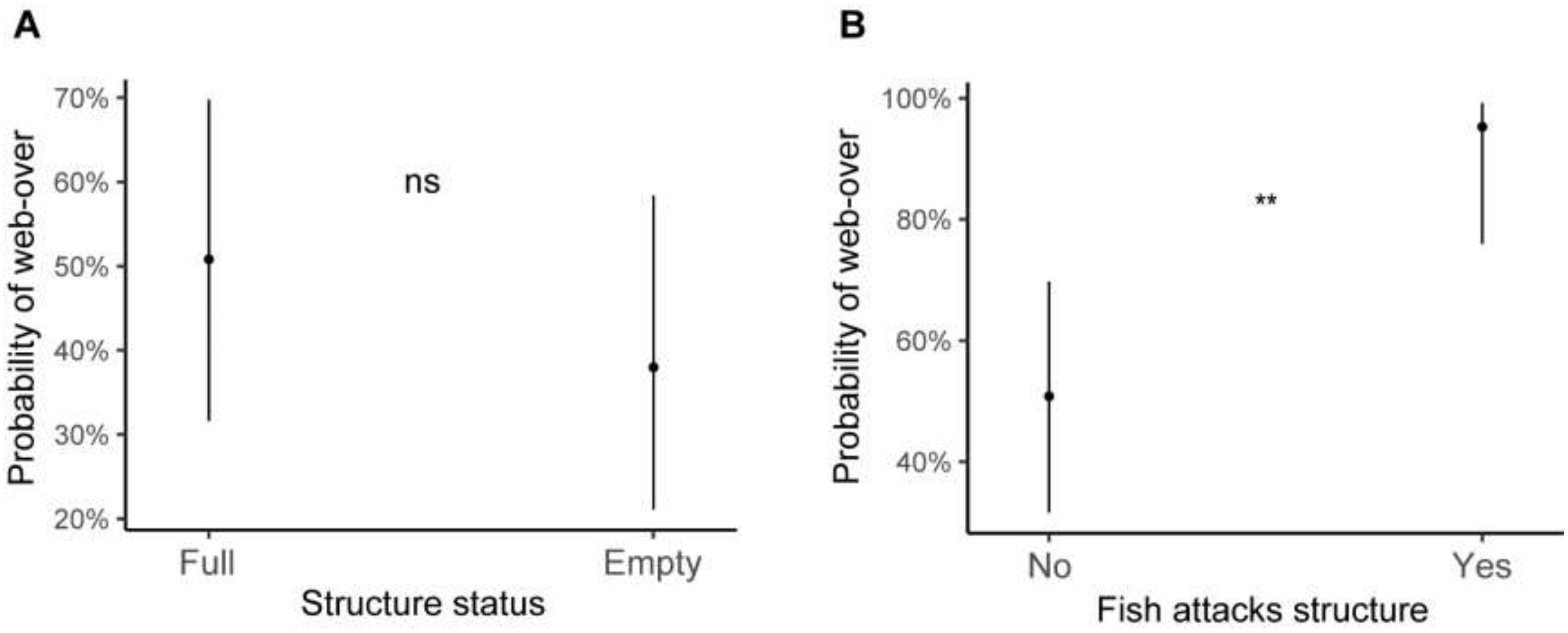

Supplementary Figure 17. Web-over duration is independent of fish presence. Experimental results demonstrating that duration of web-overs is independent of fish presence (no significant differences), varying only due to food status (empty or full) of the structure (Supplementary Table 44, Fig. 5b). Data points, violin plots, and boxplots are used. For boxplots, boxes represent the 25% and 75% quartiles, with the center (50%) being the median, and red dots indicating the mean. Whiskers represent the equal or lower/upper value of 1.5 * the interquartile range (between 25-75%).

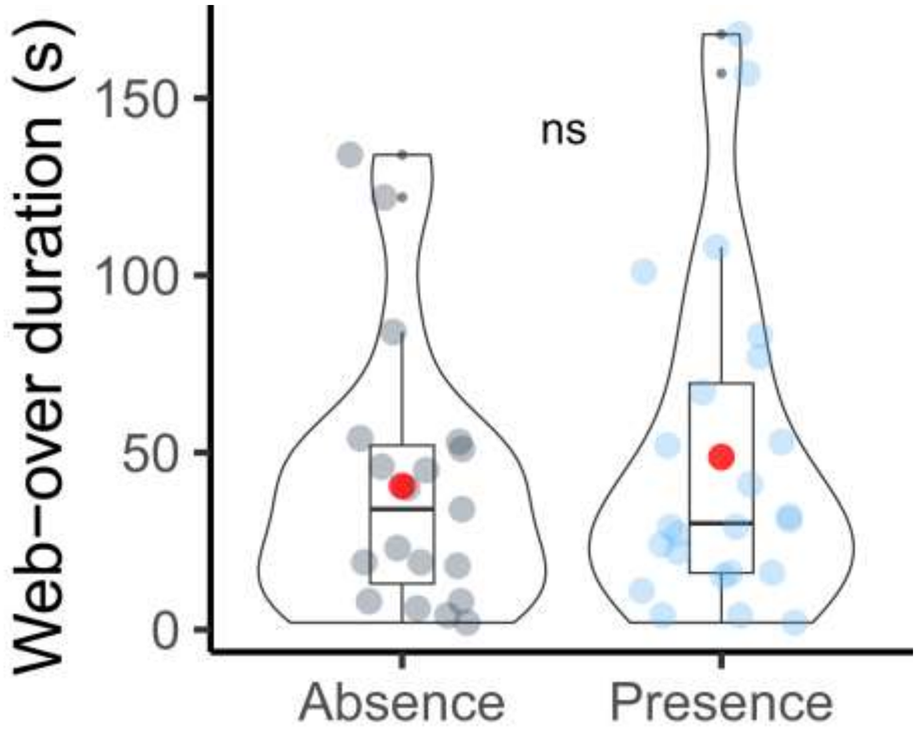

Supplementary Figure 18. Controlling for hunting group block length. Boxplots and datapoints depicting species-specific performance regarding pulling and anchoring frequency per minute, depending on dividing groups into subgroups according to lengths of **(A-B)** 200 seconds (Supplementary Table 47-S48) or **(C-D)** 300 seconds (Supplementary Table 49-S50). Values were also normalized considering the number of individuals that were present in each subgroupFor boxplots, boxes represent the 25% and 75% quartiles, with the center (50%) being the median, and red dots indicating the mean. Whiskers represent the equal or lower/upper value of 1.5 * the interquartile range (between 25-75%). Different letters indicate significant differences ($p < 0.05$), see Tables S47-S50.

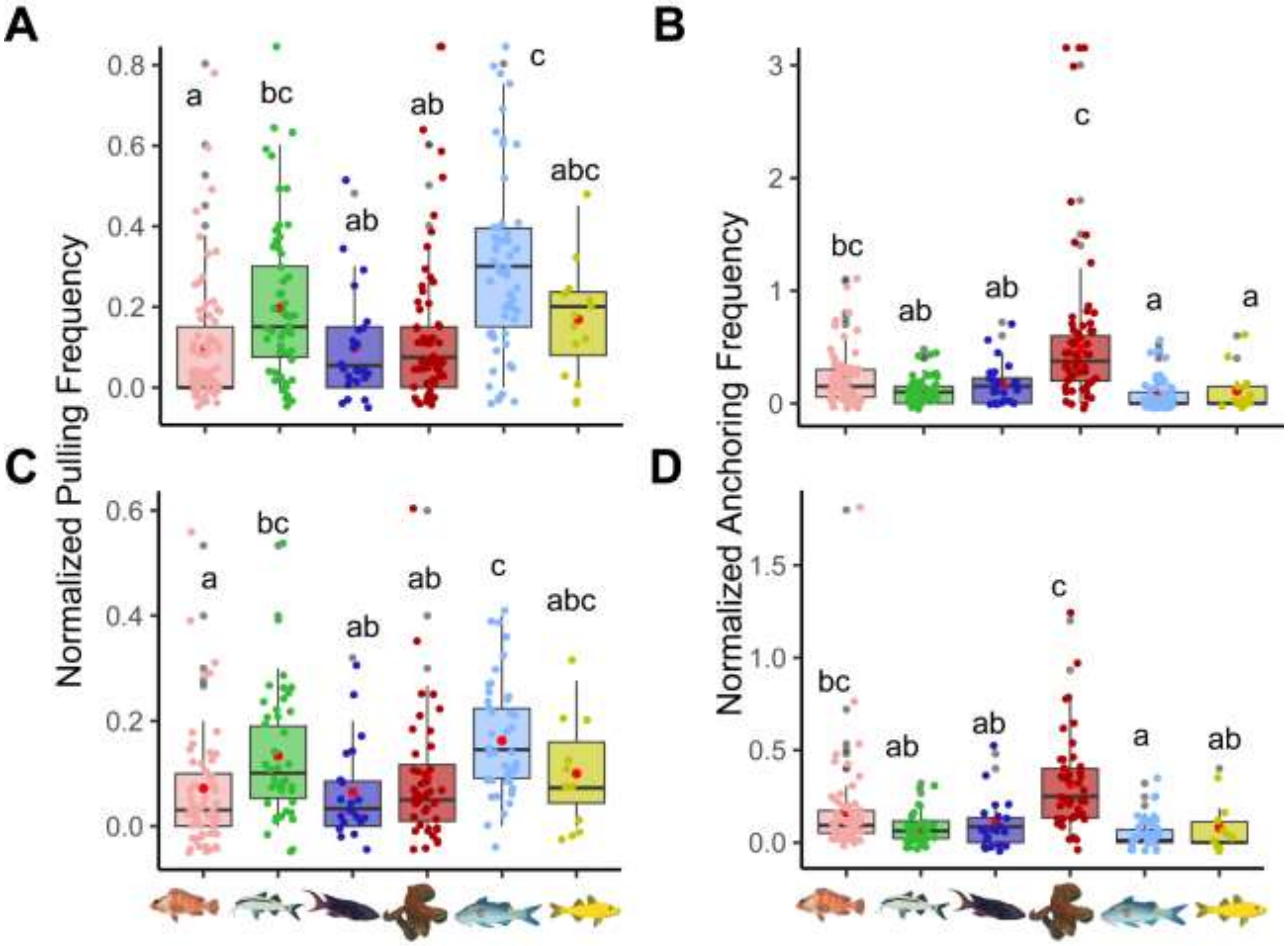

Supplementary Figure 19. Pull-Anchor Angles. Radial histograms showing the raw frequency distribution of angles between the movement vectors of the initiator (during t1-t2) and the potential follower/anchorer (during t2-t3), when initiations are successful (Pull Angles) and unsuccessful (Anchor Angles). Scale is maintained to better depict differences between the two plots.

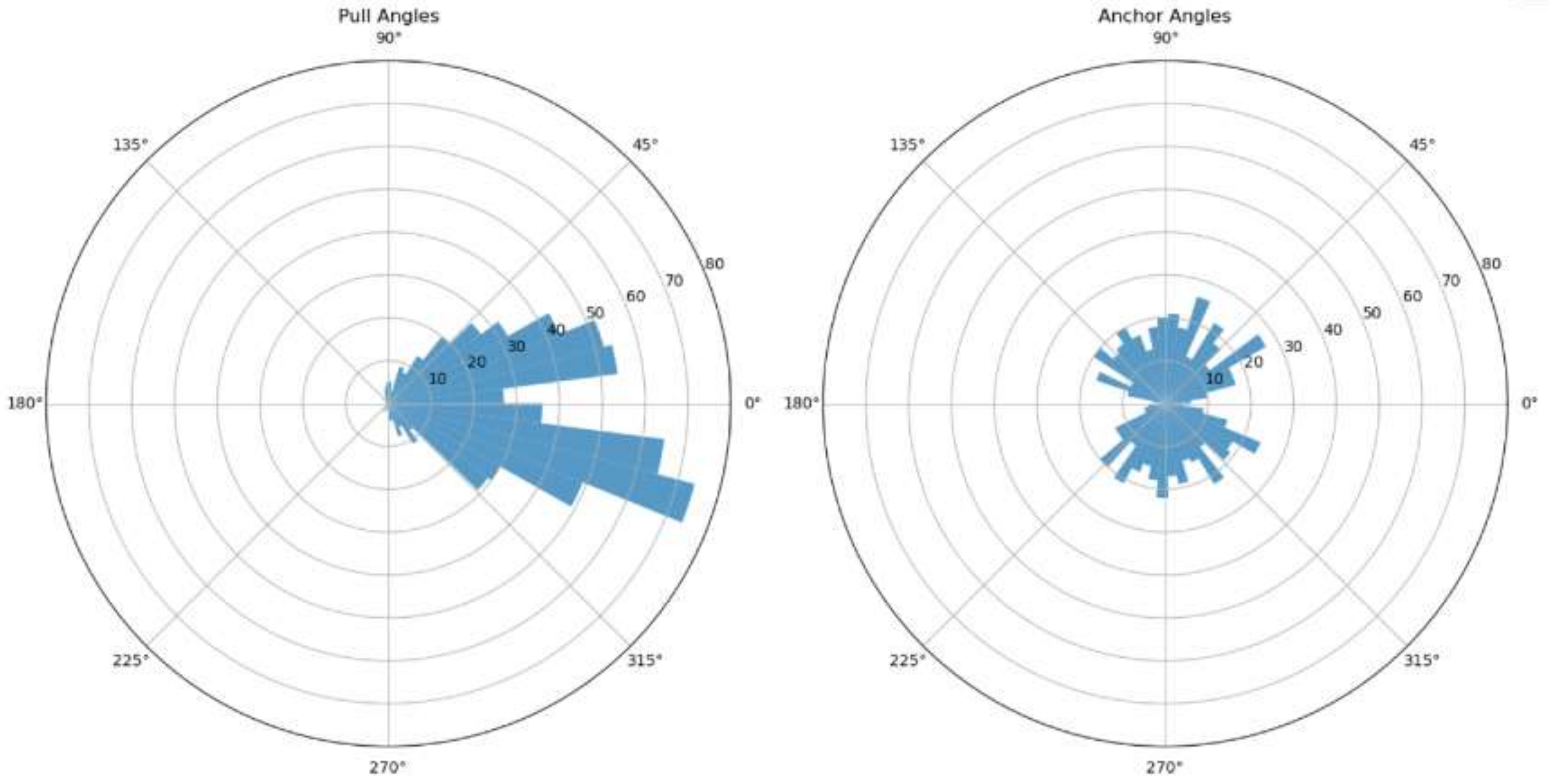

Supplementary Table 1. Common and scientific names of the species present in the study, as well as the short name we refer to in the main text. The gold-saddle goatfish, due to phenotypical differences, is split into two categories: yellow phase and blue phase.

**species name**	**latin name**	**short name**
day octopus	*Octopus cyanea*	octopus
long-barbel goatfish	*Parupeneus macronemus*	barbel goatfish
yellow phase gold-saddle goatfish	*Parupeneus cyclostomus*	yellow goatfish
blue phase gold-saddle goatfish	*Parupeneus cyclostomus*	blue goatfish
lyretail grouper	*Variola louti*	lyretail
blacktip grouper	*Epinephelus fasciatus*	blacktip

Supplementary Table 2. Generalized linear mixed effects model (GLMM) and Tukey Honest Significant Differences (Tukey HSD) pairwise comparisons assessing significant differences in the rate of pulls between phenotypes. Rates of pulls were measured as the frequency of pulls within a finite time interval (1.66 minutes, i.e. the duration of one block) and the number of individuals present in that block, minus the puller ($\boldsymbol{n-1}$). Lastly, scene (hunting group), individual identity (individual), and block (hunting subgroup) were used as a nested random effect, with the formula: *pulling ~ phenotype + (1 | scene/individual/block) + offset(log((1.66) * (n - 1)), family = poisson*. Presented are coefficient estimates, standard errors (SE), standard deviation (Std. Dev.), z-values, and p-values (Tukey-adjusted), with statistical significance shown as '****' for p < 0.0001, '***' for p < 0.001, '**' for p < 0.01, and '*' for p < 0.05.

Random effects

Groups	*Name*	*Variance*	*Std.Dev.*
block:individual:scene	(Intercept)	0.2318	0.4815
individual:scene	(Intercept)	0.0022	0.0467
scene	(Intercept)	0.1290	0.3592

Number of obs: 501, groups: block:individual:scene, 501; individual:scene, 75; scene, 13

Conditional model

	Estimate	*SE*	*z-value*	*p-value*	
(Intercept)	-2.583	0.181	-14.232	<0.0001	****
barbel goatfish	0.646	0.171	3.779	0.0002	***
lyretail	-0.052	0.257	-0.201	0.8403	
octopus	0.191	0.188	1.013	0.3113	
blue goatfish	1.127	0.183	6.146	<0.0001	****
yellow goatfish	0.815	0.274	2.969	0.0030	**

Tukey HSD Pairwise comparisons

Contrast	*Estimate*	*SE*	*z-value*	*p-value*	
blacktip - barbel goatfish	-0.473	0.142	-3.343	0.0108	**
blacktip - lyretail	0.026	0.129	0.204	1.0000	
blacktip - octopus	-0.110	0.109	-1.004	0.9167	
blacktip - blue goatfish	-1.088	0.223	-4.870	<.0001	***
blacktip - yellow goatfish	-0.656	0.296	-2.214	0.2311	
barbel goatfish - lyretail	0.499	0.174	2.866	0.0477	*
barbel goatfish - octopus	0.363	0.149	2.439	0.1428	
barbel goatfish - blue goatfish	-0.615	0.228	-2.698	0.0757	
barbel goatfish - yellow goatfish	-0.183	0.313	-0.584	0.9921	
lyretail - octopus	-0.136	0.137	-0.990	0.9213	
lyretail - blue goatfish	-1.114	0.222	-5.014	<0.0001	***
lyretail - yellow goatfish	-0.682	0.306	-2.233	0.2226	
octopus - blue goatfish	-0.978	0.217	-4.511	0.0001	***
octopus - yellow goatfish	-0.546	0.299	-1.829	0.4471	
blue goatfish - yellow goatfish	0.432	0.317	1.364	0.7489	

Supplementary Table 3. Generalized linear mixed effects model (GLMM) and Tukey Honest Significant Differences (Tukey HSD) pairwise comparisons assessing significant differences in the rate of being anchored between phenotypes. Anchored rates were measured as the frequency of anchors within a finite time interval (1.66 minutes, i.e. the duration of one block) and the number of individuals present in that block, minus the puller ($\boldsymbol{n-1}$). Lastly, scene (hunting group), individual identity (individual), and block (hunting subgroup) were used as a nested random effect, with the formula: *anchored ~ phenotype + (1 | scene/individual/block) + offset(log((1.66) * (n - 1)), family = poisson*. Presented are coefficient estimates, standard errors (SE), standard deviation (Std. Dev.), z-values, and p-values (Tukey-adjusted), with statistical significance shown as '****' for p < 0.0001, '***' for p < 0.001, '**' for p < 0.01, and '*' for p < 0.05.

Random effects

Groups	*Name*	*Variance*	*Std.Dev.*
block:individual:scene	(Intercept)	0.366	0.605
individual:scene	(Intercept)	0.236	0.486
scene	(Intercept)	0.433	0.658

Number of obs: 501, groups: block:individual:scene, 501; individual:scene, 75; scene, 13

Conditional model

	Estimate	*SE*	*z-value*	*p-value*	
(Intercept)	-3.389	0.339	-10.009	<0.0001	****
barbel goatfish	1.714	0.291	5.891	<0.0001	****
lyretail	1.241	0.475	2.613	0.0090	**
octopus	-0.090	0.361	-0.249	0.8036	
blue goatfish	2.425	0.392	6.188	<0.0001	****
yellow goatfish	2.716	0.395	6.882	<0.0001	****

Tukey HSD Pairwise comparisons

Contrast	*Estimate*	*SE*	*z-value*	*p-value*	
blacktip - barbel goatfish	-1.060	0.359	-2.951	0.0373	*
blacktip - lyretail	-0.573	0.312	-1.833	0.4446	
blacktip - octopus	0.020	0.080	0.249	0.9999	
blacktip - blue goatfish	-2.399	0.702	-3.417	0.0083	**
blacktip - yellow goatfish	-3.287	1.057	-3.111	0.0230	*
barbel goatfish - lyretail	0.488	0.462	1.056	0.8988	
barbel goatfish - octopus	1.080	0.375	2.883	0.0455	*
barbel goatfish - blue goatfish	-1.338	0.732	-1.828	0.4474	
barbel goatfish - yellow goatfish	-2.227	1.031	-2.160	0.2566	
lyretail - octopus	0.593	0.305	1.943	0.3758	
lyretail - blue goatfish	-1.826	0.634	-2.879	0.0461	*
lyretail - yellow goatfish	-2.714	1.037	-2.618	0.0929	
octopus - blue goatfish	-2.419	0.693	-3.488	0.0065	**
octopus - yellow goatfish	-3.307	1.063	-3.112	0.0229	*
blue goatfish - yellow goatfish	-0.888	0.887	-1.002	0.9175	

Supplementary Table 4. Generalized linear mixed effects model (GLMM) and Tukey Honest Significant Differences (Tukey HSD) pairwise comparisons assessing significant differences in the rate of initiations between phenotypes. Rates of initiations were measured as the frequency of initiations within a finite time interval (1.66 minutes, i.e. the duration of one block) and the number of individuals present in that block, minus the puller ($\boldsymbol{n-1}$). Lastly, scene (hunting group), individual identity (individual), and block (hunting subgroup) were used as a nested random effect, with the formula: *initiations ~ phenotype + (1 | scene/individual/block) + offset(log((1.66) * (n - 1)), family = nbinom1*. Presented are coefficient estimates, standard errors (SE), standard deviation (Std. Dev.), z-values, and p-values (Tukey-adjusted), with statistical significance shown as '****' for p < 0.0001, '***' for p < 0.001, '**' for p < 0.01, and '*' for p < 0.05.

Random effects

Groups	***Name***	***Variance***	***Std.Dev.***
block:individual:scene	(Intercept)	0.073	0.270
individual:scene	(Intercept)	0.001	0.030
scene	(Intercept)	0.164	0.404

Number of obs: 501, groups: block:individual:scene, 501; individual:scene, 75; scene, 13

Conditional model

	Estimate	***SE***	***z-value***	***p-value***	
(Intercept)	-2.156	0.184	-11.728	<0.0001	****
barbel goatfish	1.270	0.139	9.149	<0.0001	****
lyretail	0.671	0.246	2.732	0.0063	****
octopus	0.303	0.172	1.755	0.0792	
blue goatfish	1.683	0.179	9.424	<0.0001	****
yellow goatfish	1.751	0.233	7.517	<0.0001	****

Tukey HSD Pairwise comparisons

Contrast	***Estimate***	***SE***	***z-value***	***p-value***	
blacktip - barbel goatfish	-2.044	0.369	-5.545	<0.0001	****
blacktip - lyretail	-0.764	0.345	-2.218	0.2291	
blacktip - octopus	-0.282	0.166	-1.695	0.5352	
blacktip - blue goatfish	-3.499	0.603	-5.805	<0.0001	****
blacktip - yellow goatfish	-3.802	0.933	-4.075	0.0007	***
barbel goatfish - lyretail	1.280	0.438	2.923	0.0406	*
barbel goatfish - octopus	1.762	0.362	4.872	<0.0001	****
barbel goatfish - blue goatfish	-1.454	0.548	-2.656	0.0843	
barbel goatfish - yellow goatfish	-1.758	0.900	-1.952	0.3704	
lyretail - octopus	0.482	0.326	1.479	0.678	
lyretail - blue goatfish	-2.734	0.534	-5.117	<0.0001	****
lyretail - yellow goatfish	-3.038	0.891	-3.409	0.0086	**
octopus - blue goatfish	-3.217	0.570	-5.646	<0.0001	****
octopus - yellow goatfish	-3.520	0.911	-3.866	0.0015	**
blue goatfish - yellow goatfish	-0.303	0.814	-0.372	0.9991	

Supplementary Table 5. Generalized linear mixed effects model (GLMM) and Tukey Honest Significant Differences (Tukey HSD) pairwise comparisons assessing significant differences in the rate of following initiations between phenotypes. Rates of following initiations were measured as the frequency of following within a finite time interval (1.66 minutes, i.e. the duration of one block) and the number of individuals present in that block, minus the puller ($\boldsymbol{n-1}$). Lastly, scene (hunting group), individual identity (individual), and block (hunting subgroup) were used as a nested random effect, with the formula: *follower ~ phenotype + (1 | scene/individual/block) + offset(log((1.66) * (n - 1)), family = poisson*. Presented are coefficient estimates, standard errors (SE), standard deviation (Std. Dev.), z-values, and p-values (Tukey-adjusted), with statistical significance shown as '****' for p < 0.0001, '***' for p < 0.001, '**' for p < 0.01, and '*' for p < 0.05.

Random effects

Groups	*Name*	*Variance*	*Std.Dev.*
block:individual:scene	(Intercept)	0.269	0.519
individual:scene	(Intercept)	0.000	0.000
scene	(Intercept)	0.189	0.435

Number of obs: 501, groups: block:individual:scene, 501; individual:scene, 75; scene, 13

Conditional model

	Estimate	*SE*	*z-value*	*p-value*	
(Intercept)	-2.136	0.189	-11.327	<0.0001	****
barbel goatfish	0.241	0.162	1.489	0.1360	
lyretail	0.271	0.218	1.242	0.2140	
octopus	0.168	0.169	0.991	0.3220	
blue goatfish	-0.037	0.193	-0.192	0.8470	
yellow goatfish	-0.071	0.294	-0.243	0.8080	

Tukey HSD Pairwise comparisons

Contrast	*Estimate*	*SE*	*z-value*	*p-value*
blacktip - barbel goatfish	-0.222	0.153	-1.449	0.6968
blacktip - lyretail	-0.254	0.216	-1.177	0.8482
blacktip - octopus	-0.149	0.150	-0.990	0.9212
blacktip - blue goatfish	0.030	0.155	0.192	1.0000
blacktip - yellow goatfish	0.056	0.227	0.247	0.9999
barbel goatfish - lyretail	-0.032	0.224	-0.140	1.0000
barbel goatfish - octopus	0.073	0.168	0.436	0.9980
barbel goatfish - blue goatfish	0.252	0.175	1.440	0.7028
barbel goatfish - yellow goatfish	0.278	0.246	1.129	0.8694
lyretail - octopus	0.105	0.209	0.503	0.9961
lyretail - blue goatfish	0.283	0.197	1.438	0.7036
lyretail - yellow goatfish	0.310	0.268	1.155	0.8579
octopus - blue goatfish	0.179	0.150	1.194	0.8401
octopus - yellow goatfish	0.205	0.228	0.898	0.9470
blue goatfish - yellow goatfish	0.026	0.214	0.124	1.0000

Supplementary Table 6. Generalized linear mixed effects model (GLMM) and Tukey Honest Significant Differences (Tukey HSD) pairwise comparisons assessing significant differences in the rate of anchoring between phenotypes. Rates of anchoring others were measured as the frequency of anchoring within a finite time interval (1.66 minutes, i.e. the duration of one block) and the number of individuals present in that block, minus the puller ($\boldsymbol{n-1}$). Lastly, scene (hunting group), individual identity (individual), and block (hunting subgroup) were used as a nested random effect, with the formula: *anchoring ~ phenotype + (1 | scene/individual/block) + offset(log((1.66) * (n - 1)), family = nbinom1*. Presented are coefficient estimates, standard errors (SE), standard deviation (Std. Dev.), z-values, and p-values (Tukey-adjusted), with statistical significance shown as '****' for p < 0.0001, '***' for p < 0.001, '**' for p < 0.01, and '*' for p < 0.05.

Random effects

Groups	*Name*	*Variance*	*Std.Dev.*
block:individual:scene	(Intercept)	0.004	0.064
individual:scene	(Intercept)	0.071	0.266
scene	(Intercept)	0.094	0.307

Number of obs: 357, groups: block:individual:scene, 357; individual:scene, 69; scene, 13

Conditional model

	Estimate	*SE*	*z-value*	*p-value*	
(Intercept)	-1.750	0.174	-10.072	<0.0001	****
barbel goatfish	-0.344	0.211	-1.633	0.1025	
lyretail	-0.116	0.288	-0.405	0.6858	
octopus	0.790	0.177	4.464	<0.0001	****
blue goatfish	-0.891	0.253	-3.526	0.0004	***
yellow goatfish	-0.958	0.410	-2.335	0.0195	*

Tukey HSD Pairwise comparisons

Contrast	*Estimate*	*SE*	*z-value*	*p-value*	
blacktip - barbel goatfish	0.349	0.209	1.668	0.5531	
blacktip - lyretail	0.132	0.316	0.416	0.9984	
blacktip - octopus	-1.442	0.378	-3.818	0.0019	**
blacktip - blue goatfish	0.707	0.208	3.402	0.0088	**
blacktip - yellow goatfish	0.739	0.255	2.901	0.0432	*
barbel goatfish - lyretail	-0.217	0.306	-0.710	0.9808	
barbel goatfish - octopus	-1.791	0.385	-4.652	<.0001	***
barbel goatfish - blue goatfish	0.358	0.187	1.915	0.3928	
barbel goatfish - yellow goatfish	0.390	0.242	1.608	0.5934	
lyretail - octopus	-1.574	0.436	-3.611	0.0041	**
lyretail - blue goatfish	0.575	0.283	2.034	0.3228	
lyretail - yellow goatfish	0.607	0.328	1.849	0.4340	
octopus - blue goatfish	2.149	0.392	5.488	<0.0001	****
octopus - yellow goatfish	2.181	0.424	5.138	<0.0001	****
blue goatfish - yellow goatfish	0.032	0.200	0.161	1.0000	

Supplementary Table 7. Generalized linear mixed effects model (GLMM) and Tukey Honest Significant Differences (Tukey HSD) pairwise comparisons assessing significant differences in pulling efficiency between phenotypes. Efficiencies in pulling others were calculated as the frequency of pulling weighted by the frequency of initiations. Lastly, scene (hunting group), individual identity (individual), and block (hunting subgroup) were used as a nested random effect, with the formula: *cbind(pulling,anchored) ~ phenotype + (1 | scene/individual/block)*, *family = binomial*. Presented are coefficient estimates, standard errors (SE), standard deviation (Std. Dev.), z-values, and p-values (Tukey-adjusted), with statistical significance shown as '****' for p < 0.0001, '***' for p < 0.001, '**' for p < 0.01, and '*' for p < 0.05.

Random effects

Groups	*Name*	*Variance*	*Std.Dev.*
block:individual:scene	(Intercept)	0.743	0.862
individual:scene	(Intercept)	0.325	0.570
scene	(Intercept)	0.567	0.753

Number of obs: 501, groups: block:individual:scene, 501; individual:scene, 75; scene, 13

Conditional model

	Estimate	*SE*	*z-value*	*p-value*	
(Intercept)	0.7434	0.4043	1.839	0.0660	.
barbel goatfish	-1.053	0.393	-2.682	0.0073	**
lyretail	-1.451	0.611	-2.377	0.0175	*
octopus	0.623	0.494	1.260	0.2075	
blue goatfish	-1.040	0.460	-2.262	0.0237	*
yellow goatfish	-1.690	0.598	-2.824	0.0047	**

Tukey HSD Pairwise comparisons

Contrast	*Estimate*	*SE*	*z-value*	*p-value*	
blacktip - barbel goatfish	0.254	0.090	2.822	0.0540	
blacktip - lyretail	0.348	0.134	2.587	0.1005	
blacktip - octopus	-0.119	0.094	-1.273	0.8001	
blacktip - blue goatfish	0.251	0.105	2.389	0.1599	
blacktip - yellow goatfish	0.398	0.124	3.207	0.0169	*
barbel goatfish - lyretail	0.093	0.131	0.713	0.9805	
barbel goatfish - octopus	-0.374	0.094	-3.984	0.0010	**
barbel goatfish - blue goatfish	-0.003	0.102	-0.030	1.0000	
barbel goatfish - yellow goatfish	0.144	0.125	1.149	0.8606	
lyretail - octopus	-0.467	0.125	-3.729	0.0026	**
lyretail - blue goatfish	-0.096	0.114	-0.843	0.9594	
lyretail - yellow goatfish	0.050	0.142	0.355	0.9993	
octopus - blue goatfish	0.370	0.093	3.979	0.0010	**
octopus - yellow goatfish	0.517	0.118	4.389	0.0002	***
blue goatfish - yellow goatfish	0.147	0.110	1.336	0.7651	

Supplementary Table 8. Generalized linear mixed effects model (GLMM) and Tukey Honest Significant Differences (Tukey HSD) pairwise comparisons assessing significant differences in anchoring efficiency between phenotypes. Efficiency in anchoring others were calculated as the frequency of anchoring weighted by the frequency of opportunities to do so, or follow. In this case, an autocorrelation structure based on incremental ‘blocks’ was successfully fitted (Corr), with scene (hunting group) and individual identity (individual) used as a nested random effect, in the formula: *cbind(anchoring,follower) ~ phenotype + ar1(block + 0 | scene/individual), family = binomial*. Presented are coefficient estimates, standard errors (SE), standard deviation (Std. Dev.), z-values, and p-values (Tukey-adjusted), with statistical significance shown as '****' for p < 0.0001, '***' for p < 0.001, '**' for p < 0.01, and '*' for p < 0.05.

Random effects

Groups	*Name*	*Variance*	*Std.Dev.*	*Corr*
individual:scene	block1	0.24	0.4899	-0.18 (ar1)
scene	block1	0.7938	0.891	0.69 (ar1)

Number of obs: 501, groups: block:individual:scene, 501; individual:scene, 75; scene, 13

Conditional model

	Estimate	*SE*	*z-value*	*p-value*	
(Intercept)	0.3248	0.2445	1.329	0.1840	
barbel goatfish	-0.6924	0.237	-2.922	0.0035	**
lyretail	-0.3674	0.3279	-1.121	0.2625	
octopus	0.6209	0.2166	2.867	0.0041	**
blue goatfish	-1.0405	0.295	-3.527	0.0004	***
yellow goatfish	-0.9047	0.4654	-1.944	0.0519	

Tukey HSD Pairwise comparisons

Contrast	*Estimate*	*SE*	*z-value*	*p-value*	
blacktip - barbel goatfish	0.171	0.058	2.982	0.0341	*
blacktip - lyretail	0.091	0.081	1.121	0.8728	
blacktip - octopus	-0.140	0.050	-2.800	0.0574	
blacktip - blue goatfish	0.252	0.068	3.717	0.0028	**
blacktip - yellow goatfish	0.222	0.108	2.049	0.3146	
barbel goatfish - lyretail	-0.080	0.083	-0.967	0.9285	
barbel goatfish - octopus	-0.311	0.054	-5.716	<0.0001	***
barbel goatfish - blue goatfish	0.081	0.070	1.161	0.8555	
barbel goatfish - yellow goatfish	0.050	0.110	0.458	0.9975	
lyretail - octopus	-0.231	0.074	-3.116	0.0226	*
lyretail - blue goatfish	0.161	0.079	2.051	0.3137	
lyretail - yellow goatfish	0.130	0.115	1.131	0.8687	
octopus - blue goatfish	0.392	0.058	6.726	<0.0001	****
octopus - yellow goatfish	0.361	0.102	3.539	0.0054	**
blue goatfish - yellow goatfish	-0.031	0.103	-0.297	0.9997	

Supplementary Table 9. Generalized linear mixed effects model (GLMM) and Tukey Honest Significant Differences (Tukey HSD) pairwise comparisons assessing significant differences in pulling/follower ratios between phenotypes. The ratios were calculated as the frequency of pulling divided by the frequency of being a follower within a finite time interval (1.66 minutes, i.e. the duration of one block) and the number of individuals present in that block, minus the puller ($\boldsymbol{n-1}$). An autocorrelation structure based on incremental 'blocks' was successfully fitted (Corr), with scene (hunting group) and individual identity (individual) used as a nested random effect, in the formula: *pulling ~ phenotype + ar1(block + 0 | scene/individual) + offset(log((follower + 1.66) * (n - 1)), family = poisson*. Presented are coefficient estimates, standard errors (SE), standard deviation (Std. Dev.), z-values, and p-values (Tukey-adjusted), with statistical significance shown as '****' for p < 0.0001, '***' for p < 0.001, '**' for p < 0.01, and '*' for p < 0.05.

Random effects

Groups	*Name*	*Variance*	*Std.Dev.*
block:individual:scene	(Intercept)	0.520	0.721
individual:scene	(Intercept)	0.045	0.213
scene	(Intercept)	0.016	0.127

Number of obs: 501, groups: block:individual:scene, 501; individual:scene, 75; scene, 13

Conditional model

	Estimate	*SE*	*z-value*	*p-value*	
(Intercept)	-2.847	0.164	-17.332	<0.0001	****
barbel goatfish	0.694	0.213	3.260	0.0011	**
lyretail	-0.214	0.298	-0.717	0.4732	
octopus	0.214	0.224	0.953	0.3405	
blue goatfish	1.216	0.208	5.854	<0.0001	****
yellow goatfish	0.741	0.346	2.139	0.0325	*

Tukey HSD Pairwise comparisons

Contrast	*Estimate*	*SE*	*z-value*	*p-value*	
blacktip - barbel goatfish	-0.490	0.168	-2.925	0.0403	*
blacktip - lyretail	0.094	0.126	0.748	0.9759	
blacktip - octopus	-0.117	0.124	-0.937	0.9370	
blacktip - blue goatfish	-1.162	0.239	-4.867	<0.0001	****
blacktip - yellow goatfish	-0.537	0.326	-1.647	0.5674	
barbel goatfish - lyretail	0.584	0.187	3.132	0.0215	*
barbel goatfish - octopus	0.374	0.181	2.067	0.3047	
barbel goatfish - blue goatfish	-0.672	0.280	-2.402	0.1556	
barbel goatfish - yellow goatfish	-0.047	0.368	-0.127	1.0000	
lyretail - octopus	-0.211	0.143	-1.472	0.6825	
lyretail - blue goatfish	-1.256	0.241	-5.212	<0.0001	****
lyretail - yellow goatfish	-0.631	0.329	-1.918	0.3912	
octopus - blue goatfish	-1.045	0.247	-4.239	0.0003	***
octopus - yellow goatfish	-0.420	0.334	-1.257	0.8084	
blue goatfish - yellow goatfish	0.625	0.362	1.724	0.5154	

Supplementary Table 10. Generalized linear mixed effects model (GLMM) and Tukey Honest Significant Differences (Tukey HSD) pairwise comparisons assessing significant differences in initiations/follower ratios between phenotypes. The ratios were calculated as the frequency of initiations divided by the frequency of being a follower within a finite time interval (1.66 minutes, i.e. the duration of one block) and the number of individuals present in that block, minus the puller ($\boldsymbol{n-1}$). An autocorrelation structure based on incremental 'blocks' was successfully fitted (Corr), with scene (hunting group) and individual identity (individual) used as a nested random effect, in the formula: *initiations ~ phenotype + ar1(block + 0 | scene/individual) + offset(log((follower + 1.66) * (n - 1)), family = nbinom2*. Presented are coefficient estimates, standard errors (SE), standard deviation (Std. Dev.), z-values, and p-values (Tukey-adjusted), with statistical significance shown as '****' for p < 0.0001, '***' for p < 0.001, '**' for p < 0.01, and '*' for p < 0.05.

Random effects

Groups	*Name*	*Variance*	*Std.Dev.*	*Corr*
individual:scene	block1	0.280	0.529	0.74 (ar1)
scene	block1	0.179	0.424	0.99 (ar1)

Number of obs: 501, groups: block:individual:scene, 501; individual:scene, 75; scene, 13

Conditional model

	Estimate	*SE*	*z-value*	*p-value*	
(Intercept)	-2.227	0.218	-10.232	<0.0001	***
barbel goatfish	1.301	0.209	6.241	<0.0001	***
lyretail	0.363	0.351	1.032	0.3020	
octopus	0.157	0.235	0.668	0.5040	
blue goatfish	1.800	0.273	6.588	<0.0001	***
yellow goatfish	1.833	0.384	4.780	<0.0001	***

Tukey HSD Pairwise comparisons

Contrast	*Estimate*	*SE*	*z-value*	*p-value*	
blacktip - barbel goatfish	-2.433	0.627	-3.878	0.0015	**
blacktip - lyretail	-0.398	0.422	-0.943	0.9354	
blacktip - octopus	-0.155	0.233	-0.663	0.9859	
blacktip - blue goatfish	-4.593	1.137	-4.041	0.0008	***
blacktip - yellow goatfish	-4.782	1.930	-2.477	0.1307	
barbel goatfish - lyretail	2.035	0.762	2.670	0.0814	
barbel goatfish - octopus	2.278	0.652	3.495	0.0063	**
barbel goatfish - blue goatfish	-2.161	1.230	-1.757	0.4938	
barbel goatfish - yellow goatfish	-2.350	2.008	-1.170	0.8512	
lyretail - octopus	0.243	0.409	0.595	0.9914	
lyretail - blue goatfish	-4.195	1.052	-3.989	0.0009	***
lyretail - yellow goatfish	-4.384	1.876	-2.337	0.1791	
octopus - blue goatfish	-4.439	1.105	-4.017	0.0008	***
octopus - yellow goatfish	-4.628	1.912	-2.420	0.1491	
blue goatfish - yellow goatfish	-0.189	1.831	-0.103	1	

Supplementary Table 11. Generalized linear mixed effects model (GLMM) and Tukey Honest Significant Differences (Tukey HSD) pairwise comparisons assessing significant differences in the rate of first pulls between phenotypes. Rates of first pulls were measured as the frequency of being the first individual to pull another, within a finite time interval (1.66 minutes, i.e. the duration of one block) and the number of individuals present in that block, minus the puller ($\boldsymbol{n-1}$). Lastly, scene (hunting group), individual identity (individual), and block (hunting subgroup) were used as a nested random effect, with the formula: 1st *pulling ~ phenotype + (1 | scene/individual/block) + offset(log((1.66) * (n - 1)), family = poisson*. Presented are coefficient estimates, standard errors (SE), standard deviation (Std. Dev.), z-values, and p-values (Tukey-adjusted), with statistical significance shown as '****' for p < 0.0001, '***' for p < 0.001, '**' for p < 0.01, and '*' for p < 0.05.

Random effects

Groups	***Name***	***Variance***	***Std.Dev.***
block:individual:scene	(Intercept)	0.2998	0.5475
individual:scene	(Intercept)	0.0309	0.1758
scene	(Intercept)	0.0451	0.2122

Number of obs: 501, groups: block:individual:scene, 501; individual:scene, 75; scene, 13

Conditional model

	Estimate	***SE***	***z-value***	***p-value***	
(Intercept)	-2.873	0.181	-15.874	<0.0001	****
barbel goatfish	0.775	0.207	3.751	0.0002	***
lyretail	-0.158	0.311	-0.507	0.6125	
octopus	0.261	0.223	1.169	0.2423	
blue goatfish	1.071	0.207	5.180	<0.0001	****
yellow goatfish	0.513	0.327	1.572	0.1160	

Tukey HSD Pairwise comparisons

Contrast	***Estimate***	***SE***	***z-value***	***p-value***	
blacktip - barbel goatfish	-0.457	0.136	-3.363	0.0100	*
blacktip - lyretail	0.057	0.109	0.524	0.9953	
blacktip - octopus	-0.116	0.101	-1.150	0.8605	
blacktip - blue goatfish	-0.748	0.167	-4.475	0.0001	***
blacktip - yellow goatfish	-0.262	0.199	-1.317	0.7759	
barbel goatfish - lyretail	0.514	0.157	3.271	0.0137	*
barbel goatfish - octopus	0.341	0.146	2.334	0.1805	
barbel goatfish - blue goatfish	-0.292	0.196	-1.486	0.6730	
barbel goatfish - yellow goatfish	0.195	0.231	0.845	0.9591	
lyretail - octopus	-0.173	0.122	-1.423	0.7130	
lyretail - blue goatfish	-0.805	0.173	-4.653	<.0001	****
lyretail - yellow goatfish	-0.318	0.210	-1.513	0.6556	
octopus - blue goatfish	-0.632	0.172	-3.679	0.0032	**
octopus - yellow goatfish	-0.146	0.208	-0.701	0.9819	
blue goatfish - yellow goatfish	0.487	0.233	2.085	0.2950	

Supplementary Table 12. Generalized linear mixed effects model (GLMM) and Tukey Honest Significant Differences (Tukey HSD) pairwise comparisons assessing significant differences in the rate of pulls between phenotypes, removing pulls effectuated between same-species individuals. Rates of pulls were measured as the frequency of pulls within a finite time interval (1.66 minutes, i.e. the duration of one block) and the number of individuals present in that block, minus the puller ($\boldsymbol{n-1}$). Lastly, scene (hunting group), individual identity (individual), and block (hunting subgroup) were used as a nested random effect, with the formula: *pulling ~ phenotype + (1 | scene/individual/block) + offset(log((1.66) * (n - 1)), family = poisson*. Presented are coefficient estimates, standard errors (SE), standard deviation (Std. Dev.), z-values, and p-values (Tukey-adjusted), with statistical significance shown as '****' for p < 0.0001, '***' for p < 0.001, '**' for p < 0.01, and '*' for p < 0.05.

Random effects

Groups	*Name*	*Variance*	*Std.Dev.*
block:individual:scene	(Intercept)	0.2257	0.4751
individual:scene	(Intercept)	0.0000	0.0001
scene	(Intercept)	0.1218	0.3490

Number of obs: 501, groups: block:individual:scene, 501; individual:scene, 75; scene, 13

Conditional model

	Estimate	*SE*	*z-value*	*p-value*	
(Intercept)	-2.574	0.191	-13.475	<0.0001	****
barbel goatfish	0.612	0.182	3.367	0.0008	***
lyretail	-0.047	0.270	-0.175	0.8608	
octopus	0.161	0.196	0.823	0.4105	
blue goatfish	1.102	0.195	5.645	<0.0001	****
yellow goatfish	0.840	0.287	2.925	0.0034	**

Tukey HSD Pairwise comparisons

Contrast	*Estimate*	*SE*	*z-value*	*p-value*	
blacktip - barbel goatfish	-0.366	0.118	-3.1010	0.0237	*
blacktip - lyretail	0.020	0.113	0.1770	1.0000	
blacktip - octopus	-0.076	0.092	-0.8260	0.9629	
blacktip - blue goatfish	-0.872	0.188	-4.6290	0.0001	***
blacktip - yellow goatfish	-0.570	0.258	-2.2130	0.2317	
barbel goatfish - lyretail	0.386	0.142	2.7150	0.0723	
barbel goatfish - octopus	0.290	0.122	2.3880	0.1605	
barbel goatfish - blue goatfish	-0.506	0.190	-2.6630	0.0828	
barbel goatfish - yellow goatfish	-0.205	0.268	-0.7650	0.9734	
lyretail - octopus	-0.096	0.113	-0.8470	0.9585	
lyretail - blue goatfish	-0.892	0.187	-4.7670	<.0001	****
lyretail - yellow goatfish	-0.590	0.264	-2.2380	0.2202	
octopus - blue goatfish	-0.797	0.182	-4.3740	0.0002	***
octopus - yellow goatfish	-0.495	0.256	-1.9310	0.3829	
blue goatfish - yellow goatfish	0.432	0.317	1.364	0.7489	

Supplementary Table 13. Generalized linear mixed effects model (GLMM) and Tukey Honest Significant Differences (Tukey HSD) pairwise comparisons assessing significant differences in proportion of time not moving, between phenotypes. The ratios were calculated as the proportion of time moving at speeds < 1 cm s^{-1}, within a finite time interval (1.66 minutes, i.e. the duration of one block). An autocorrelation structure based on incremental 'blocks' was successfully fitted (Corr), with scene (hunting group) and individual identity (individual) used as a nested random effect, in the formula: *zero movement ~ phenotype + ar1(block + 0 | scene/individual), family = beta_family*. Presented are coefficient estimates, standard errors (SE), standard deviation (Std. Dev.), z-values, and p-values (Tukey-adjusted), with statistical significance shown as '****' for p < 0.0001, '***' for p < 0.001, '**' for p < 0.01, and '*' for p < 0.05.

Random effects

Groups	*Name*	*Variance*	*Std.Dev.*	*Corr*
individual:scene	block1	0.13	0.36	0.66 (ar1)
scene	block1	0.32	0.56	0.44 (ar1)

Number of obs: 366, groups: block:individual:scene, 366; individual:scene, 71; scene, 13

Conditional model

	Estimate	*SE*	*z-value*	*p-value*	
(Intercept)	-0.301	0.162	-1.855	0.0636	
barbel goatfish	-1.099	0.203	-5.405	<0.0001	****
lyretail	-0.781	0.275	-2.837	0.0046	*
octopus	-0.953	0.218	-4.376	<0.0001	****
blue goatfish	-0.932	0.229	-4.067	<0.0001	****
yellow goatfish	-0.333	0.322	-1.033	0.3017	

Tukey HSD Pairwise comparisons

Contrast	*Estimate*	*SE*	*z-value*	*p-value*	
blacktip - barbel goatfish	0.228	0.041	5.618	<0.0001	****
blacktip - lyretail	0.172	0.057	3.012	0.0312	*
blacktip - octopus	0.203	0.045	4.505	0.0001	***
blacktip - blue goatfish	0.200	0.048	4.172	0.0004	***
blacktip - yellow goatfish	0.079	0.074	1.059	0.8974	
barbel goatfish - lyretail	-0.055	0.050	-1.100	0.8815	
barbel goatfish - octopus	-0.024	0.037	-0.649	0.9872	
barbel goatfish - blue goatfish	-0.028	0.040	-0.705	0.9814	
barbel goatfish - yellow goatfish	-0.149	0.071	-2.108	0.2832	
lyretail - octopus	0.031	0.048	0.652	0.9869	
lyretail - blue goatfish	0.027	0.044	0.619	0.9897	
lyretail - yellow goatfish	-0.094	0.074	-1.271	0.8008	
octopus - blue goatfish	-0.004	0.036	-0.100	1.0000	
octopus - yellow goatfish	-0.125	0.069	-1.810	0.4589	
blue goatfish - yellow goatfish	-0.121	0.066	-1.844	0.4372	

Supplementary Table 14. Generalized linear mixed effects model (GLMM) and Tukey Honest Significant Differences (Tukey HSD) pairwise comparisons assessing significant differences in general influence between phenotypes. General influences were calculated as the sum of pulling and anchoring, within a finite time interval (1.66 minutes, i.e. the duration of one block) and the number of individuals present in that block, minus the puller ($\boldsymbol{n-1}$). An autocorrelation structure based on incremental 'blocks' was successfully fitted (Corr), with scene (hunting group) and individual identity (individual) used as a nested random effect, in the formula: *general influence ~ phenotype + ar1(block + 0| scene/individual) + offset(log((follower + 1.66) * (n - 1)), family = poisson*. Presented are coefficient estimates, standard errors (SE), standard deviation (Std. Dev.), z-values, and p-values (Tukey-adjusted), with statistical significance shown as '****' for p < 0.0001, '***' for p < 0.001, '**' for p < 0.01, and '*' for p < 0.05.

Random effects

Groups	*Name*	*Variance*	*Std.Dev.*	*Corr*
individual:scene	(Intercept)	0.123	0.351	0.89 (ar1)
scene	(Intercept)	0.234	0.484	0.28 (ar1)

Number of obs: 501, groups: block:individual:scene, 501; individual:scene, 75; scene, 13

Conditional model

	Estimate	*SE*	*z-value*	*p-value*	
(Intercept)	-1.376	0.124	-11.064	<0.0001	****
barbel goatfish	0.064	0.158	0.408	0.6830	
lyretail	-0.170	0.220	-0.774	0.4390	
octopus	0.660	0.151	4.358	<0.0001	****
blue goatfish	0.203	0.164	1.233	0.2170	
yellow goatfish	-0.146	0.254	-0.573	0.5660	

Tukey HSD Pairwise comparisons

Contrast	*Estimate*	*SE*	*z-value*	*p-value*	
blacktip - barbel goatfish	0.938	0.148	-0.408	0.9986	
blacktip - lyretail	1.185	0.260	0.774	0.9719	
blacktip - octopus	0.517	0.078	-4.358	0.0002	***
blacktip - blue goatfish	0.817	0.134	-1.233	0.8206	
blacktip - yellow goatfish	1.157	0.294	0.573	0.9928	
barbel goatfish - lyretail	1.264	0.290	1.024	0.9101	
barbel goatfish - octopus	0.551	0.091	-3.614	0.0041	**
barbel goatfish - blue goatfish	0.871	0.153	-0.788	0.9696	
barbel goatfish - yellow goatfish	1.234	0.328	0.792	0.9690	
lyretail - octopus	0.436	0.094	-3.845	0.0017	**
lyretail - blue goatfish	0.689	0.146	-1.757	0.4940	
lyretail - yellow goatfish	0.976	0.286	-0.083	1.0000	
octopus - blue goatfish	1.580	0.248	2.919	0.0410	**
octopus - yellow goatfish	2.238	0.566	3.184	0.0182	*
blue goatfish - yellow goatfish	1.417	0.355	1.392	0.7319	

Supplementary Table 15. Generalized linear mixed effects model (GLMM) and Tukey Honest Significant Differences (Tukey HSD) pairwise comparisons assessing significant differences in the rate of pulls of group's centroid between phenotypes. Rates of centroid pulls were measured as the frequency of pulls within a finite time interval (1.66 minutes, i.e. the duration of one block). Lastly, scene (hunting group) and block (hunting subgroup) were used as a nested random effect, with the formula: *centroid pulling ~ phenotype + (1 | scene/block), family = compois*. Presented are coefficient estimates, standard errors (SE), standard deviation (Std. Dev.), z-values, and p-values (Tukey-adjusted), with statistical significance shown as '****' for p < 0.0001, '***' for p < 0.001, '**' for p < 0.01, and '*' for p < 0.05.

Random effects

Groups	*Name*	*Variance*	*Std.Dev.*
block:scene	(Intercept)	0.6395	0.7997
scene	(Intercept)	0.0000	0.0000

Number of obs: 501, groups: block:individual:scene, 501; individual:scene, 75; scene, 13

Conditional model

	Estimate	*SE*	*z-value*	*p-value*	
(Intercept)	-2.314	0.271	-8.527	<0.0001	****
barbel goatfish	0.610	0.313	1.948	0.0514	***
lyretail	-0.585	0.628	-0.930	0.3522	
octopus	0.314	0.326	0.963	0.3356	
blue goatfish	1.168	0.304	3.848	0.0001	****
yellow goatfish	0.489	0.507	0.965	0.3347	**

Tukey HSD Pairwise comparisons

Contrast	*Estimate*	*SE*	*z-value*	*p-value*	
blacktip - barbel goatfish	-0.083	0.045	-1.856	0.4299	
blacktip - lyretail	0.044	0.041	1.079	0.8900	
blacktip - octopus	-0.037	0.038	-0.955	0.9318	
blacktip - blue goatfish	-0.219	0.063	-3.495	0.0063	**
blacktip - yellow goatfish	-0.062	0.075	-0.828	0.9625	
barbel goatfish - lyretail	0.127	0.051	2.492	0.1264	
barbel goatfish - octopus	0.047	0.046	1.021	0.9110	
barbel goatfish - blue goatfish	-0.136	0.065	-2.091	0.2917	
barbel goatfish - yellow goatfish	0.021	0.080	0.259	0.9998	
lyretail - octopus	-0.080	0.044	-1.846	0.4363	
lyretail - blue goatfish	-0.263	0.065	-4.046	0.0007	***
lyretail - yellow goatfish	-0.106	0.077	-1.370	0.7451	
octopus - blue goatfish	-0.183	0.060	-3.063	0.0266	*
octopus - yellow goatfish	-0.026	0.075	-0.344	0.9994	
blue goatfish - yellow goatfish	0.157	0.083	1.897	0.4039	

Supplementary Table 16. Generalized linear mixed effects model (GLMM) and Tukey Honest Significant Differences (Tukey HSD) pairwise comparisons assessing significant differences in the rate of anchoring the group's centroid between phenotypes. Rates of centroid anchoring were measured as the frequency of anchors within a finite time interval (1.66 minutes, i.e. the duration of one block). Lastly, scene (hunting group) and block (hunting subgroup) were used as a nested random effect, with the formula: *centroid anchoring ~ phenotype + (1 | scene/block), family = nbinom1*. Presented are coefficient estimates, standard errors (SE), standard deviation (Std. Dev.), z-values, and p-values (Tukey-adjusted), with statistical significance shown as '****' for $p < 0.0001$, '***' for $p < 0.001$, '**' for $p < 0.01$, and '*' for $p < 0.05$.

Random effects

Groups	*Name*	*Variance*	*Std.Dev.*
block:scene	(Intercept)	1.2690	1.1265
scene	(Intercept)	0.1498	0.3871

Number of obs: 501, groups: block:individual:scene, 501; individual:scene, 75; scene, 13

Conditional model

	Estimate	*SE*	*z-value*	*p-value*	
(Intercept)	-2.638	0.392	-6.736	<0.0001	****
barbel goatfish	0.495	0.404	1.225	0.2207	
lyretail	-1.532	1.080	-1.419	0.1559	
octopus	1.056	0.381	2.772	0.0056	**
blue goatfish	-0.826	0.559	-1.477	0.1397	
yellow goatfish	0.147	0.675	0.218	0.8276	

Tukey HSD Pairwise comparisons

Contrast	*Estimate*	*SE*	*z-value*	*p-value*	
blacktip - barbel goatfish	-0.046	0.040	-1.161	0.8555	
blacktip - lyretail	0.056	0.031	1.830	0.4462	
blacktip - octopus	-0.134	0.060	-2.243	0.2181	
blacktip - blue goatfish	0.040	0.029	1.405	0.7239	
blacktip - yellow goatfish	-0.011	0.054	-0.209	0.9999	
barbel goatfish - lyretail	0.102	0.043	2.384	0.1617	
barbel goatfish - octopus	-0.088	0.057	-1.562	0.6236	
barbel goatfish - blue goatfish	0.086	0.040	2.159	0.2573	
barbel goatfish - yellow goatfish	0.035	0.059	0.586	0.9920	
lyretail - octopus	-0.190	0.065	-2.922	0.0406	
lyretail - blue goatfish	-0.016	0.021	-0.764	0.9735	*
lyretail - yellow goatfish	-0.067	0.053	-1.283	0.7943	
octopus - blue goatfish	0.174	0.060	2.891	0.0445	*
octopus - yellow goatfish	0.123	0.067	1.832	0.4448	
blue goatfish - yellow goatfish	-0.052	0.050	-1.035	0.9064	

Supplementary Table 17. Generalized linear mixed effects model (GLMM) and Tukey Honest Significant Differences (Tukey HSD) pairwise comparisons assessing significant differences in initiations at phenotype-level and individual-level, depending on the presence of blacktips (BTG, 2 levels: YES or NO). Initiations were calculated within a finite time interval (1.66 minutes, i.e. the duration of one block) and the number of individuals present in that block, minus the puller ($\boldsymbol{n-1}$). Scene (hunting group), individual identity (individual), and block (hunting subgroup) were used as a nested random effect, with the formula: *initiations ~ phenotype * BTG + (1| scene/individual/block) + offset(log(1.66**(n-1))), family = nbinom1*. Presented are coefficient estimates, standard errors (SE), standard deviation (Std. Dev.), z-values, and p-values (Tukey-adjusted), with statistical significance shown as '****' for p < 0.0001, '***' for p < 0.001, '**' for p < 0.01, and '*' for p < 0.05.

Random effects

Groups	***Name***	***Variance***	***Std.Dev.***
block:individual:scene	(Intercept)	0.146	0.381
individual:scene	(Intercept)	0.000	0.000
scene	(Intercept)	0.254	0.504

Number of obs: 369, groups: block:individual:scene, 369; individual:scene, 53; scene, 13

Tukey HSD Pairwise comparisons

YES-NO contrasts for phenotypes	***Estimate***	***SE***	***z-value***	***p-value***	
barbel goatfish	0.802	0.757	1.06	0.2893	
lyretail	-0.761	0.519	-1.468	0.1420	
octopus	-0.104	0.281	-0.371	0.7110	
blue goatfish	-0.630	0.917	-0.687	0.4919	
yellow goatfish	-2.076	2.039	-1.018	0.3087	
BTG contrast for group	***Estimate***	***SE***	***z-value***	***p-value***	
YES - NO	-0.437	0.429	-1.017	0.3089	
Pairwise contrasts (BTG = No)	***Estimate***	***SE***	***z-value***	***p-value***	
barbel goatfish - lyretail	2.073	0.771	2.689	0.0556	
barbel goatfish - octopus	2.320	0.755	3.074	0.0180	**
barbel goatfish - blue goatfish	-0.862	0.828	-1.040	0.8367	
barbel goatfish - yellow goatfish	-0.708	1.169	-0.605	0.9743	
lyretail - octopus	0.248	0.360	0.688	0.9591	
lyretail - blue goatfish	-2.934	0.710	-4.133	0.0003	*
lyretail - yellow goatfish	-2.781	1.038	-2.679	0.0572	
octopus - blue goatfish	-3.182	0.703	-4.523	0.0001	***
octopus - yellow goatfish	-3.028	1.039	-2.916	0.0292	*
blue goatfish - yellow goatfish	0.154	0.902	0.170	0.9998	

Supplementary Table 18. Generalized linear mixed effects model (GLMM) and Tukey Honest Significant Differences (Tukey HSD) pairwise comparisons assessing significant differences in pulling efficiency at phenotype-level and individual-level, depending on the presence of blacktips (BTG, 2 levels: YES or NO). Efficiencies in pulling others were calculated as the frequency of pulling weighted by the frequency of initiations. Scene (hunting group), individual identity (individual), and block (hunting subgroup) were used as a nested random effect, with the formula: *cbind(pulling,anchored) ~ phenotype * BTG + (1| scene/individual/block) + offset(log(1.66**(n-1))), family = binomial*. Presented are coefficient estimates, standard errors (SE), standard deviation (Std. Dev.), z-values, and p-values (Tukey-adjusted), with statistical significance shown as '****' for p < 0.0001, '***' for p < 0.001, '**' for p < 0.01, and '*' for p < 0.05.

Random effects

Groups	***Name***	***Variance***	***Std.Dev.***
block:individual:scene	(Intercept)	0.521	0.722
individual:scene	(Intercept)	0.129	0.359
scene	(Intercept)	0.615	0.784

Number of obs: 369, groups: block:individual:scene, 369; individual:scene, 53; scene, 13

Tukey HSD Pairwise comparisons

YES-NO contrasts for phenotypes	***Estimate***	***SE***	***z-value***	***p-value***	
long-barbel	0.211	0.125	1.686	0.0919	
lyretail	0.144	0.156	0.924	0.3554	
octopus	0.334	0.114	2.915	0.0036	**
blue-GSG	0.290	0.103	2.828	0.0047	**
yellow-GSG	0.384	0.16	2.393	0.0167	*

BTG contrast for group	***Estimate***	***SE***	***z-value***	***p-value***	
YES - NO	0.345	0.086	3.993	0.0001	****

Pairwise contrasts (BTG = No)	***Estimate***	***SE***	***z-value***	***p-value***	
barbel goatfish - lyretail	0.144	0.180	0.800	0.9307	
barbel goatfish - octopus	-0.411	0.126	-3.257	0.0099	**
barbel goatfish - blue goatfish	-0.030	0.131	-0.232	0.9994	
barbel goatfish - yellow goatfish	0.043	0.179	0.240	0.9993	
lyretail - octopus	-0.554	0.154	-3.606	0.0029	**
lyretail - blue goatfish	-0.174	0.155	-1.126	0.7931	
lyretail - yellow goatfish	-0.101	0.192	-0.525	0.9849	
octopus - blue goatfish	0.380	0.089	4.249	0.0002	**
octopus - yellow goatfish	0.453	0.147	3.088	0.0172	*
blue goatfish - yellow goatfish	0.073	0.133	0.551	0.9819	

Supplementary Table 19. Generalized linear mixed effects model (GLMM) assessing significant differences in centroid displacement depending on the presence of blacktips (BTG, 2 levels: YES or NO). Displacement was calculated as the distance between the start and end positions of each block (i.e. distance travelled in 1.66 minutes). An autocorrelation structure based on incremental 'blocks' was successfully fitted (Corr), with scene (hunting group) used as a nesting random effect, in the formula: *centroid displacement ~ BTG + ar1(block + 0| scene), family = Gamma(link = log)*. Presented are coefficient estimates, standard errors (SE), standard deviation (Std. Dev.), z-values, and p-values (Tukey-adjusted), with statistical significance shown as '****' for p < 0.0001, '***' for p < 0.001, '**' for p < 0.01, and '*' for p < 0.05.

Random effects

Groups	***Name***	***Variance***	***Std.Dev.***	***Corr***
scene	block1	0.087	0.295	1 (ar1)

Number of obs: 107, groups: scene, 13

Conditional model

	Estimate	***SE***	***z-value***	***p-value***	
(Intercept)	1.756	0.109	16.113	<0.0001	****
BTG YES	-0.474	0.121	-3.905	<0.0001	****

Supplementary Table 20. Generalized linear mixed effects model (GLMM) assessing significant differences in mean distance to centroid depending on the presence of blacktips (BTG, 2 levels: YES or NO). Distance to centroid was calculated as the mean distance to the group's centroid across the block (i.e. 1.66 minutes) of all individuals ($\boldsymbol{n}$). Scene (hunting group) and block (subgroup) were used as a nested random effect, in the formula: *centroid distance ~ BTG + (1| scene/block) + offset(n), family = gaussian*. Presented are coefficient estimates, standard errors (SE), standard deviation (Std. Dev.), z-values, and p-values (Tukey-adjusted), with statistical significance shown as '****' for p < 0.0001, '***' for p < 0.001, '**' for p < 0.01, and '*' for p < 0.05.

Random effects

Groups	*Name*	*Variance*	*Std.Dev.*
block:scene	(Intercept)	0.034	0.185
scene	(Intercept)	1.776	1.333

Number of obs: 107, groups: timeblock:scene, 107; scene, 13

Conditional model

	Estimate	*SE*	*z-value*	*p-value*	
(Intercept)	-3.783	0.408	-9.282	<0.0001	****
BTG YES	-1.705	0.297	-5.747	<0.0001	****

Supplementary Table 21. Generalized linear mixed effects model (GLMM) and Tukey Honest Significant Differences (Tukey HSD) pairwise comparisons assessing significant differences in initiations at phenotype-level and individual-level, depending on the presence of blue goatfish (BG, 2 levels: YES or NO). Initiations were calculated within a finite time interval (1.66 minutes, i.e. the duration of one block) and the number of individuals present in that block, minus the puller $(\boldsymbol{n-1})$. Scene (hunting group), individual identity (individual), and block (hunting subgroup) were used as a nested random effect, with the formula: *initiations ~ phenotype * BG + (1| scene/individual/block) + offset(log(1.66**(n-1)*)), family = poisson*. Presented are coefficient estimates, standard errors (SE), standard deviation (Std. Dev.), z-values, and p-values (Tukey-adjusted), with statistical significance shown as '****' for $p < 0.0001$, '***' for $p < 0.001$, '**' for $p < 0.01$, and '*' for $p < 0.05$.

Random effects

Groups	*Name*	*Variance*	*Std.Dev.*
block:individual:scene	(Intercept)	0.417	0.646
individual:scene	(Intercept)	0.028	0.168
scene	(Intercept)	0.000	0.000

Number of obs: 334, groups: block:individual:scene, 334; individual:scene, 46; scene, 13

Tukey HSD Pairwise comparisons

YES-NO contrasts for phenotypes	*Estimate*	*SE*	*z-value*	*p-value*	
blacktip	0.307	0.187	1.644	0.1002	
barbel goatfish	2.497	0.629	3.972	0.0001	****
octopus	0.931	0.306	3.04	0.0024	**

BG contrast for group	*Estimate*	*SE*	*z-value*	*p-value*	
YES - NO	0.957	0.216	4.421	<0.0001	****

Pairwise contrasts (BG = No)	*Estimate*	*SE*	*z-value*	*p-value*	
blacktip- barbel goatfish	-3.140	0.543	-5.779	<0.0001	****
blacktip - octopus	-0.589	0.305	-1.930	0.1303	
barbel goatfish - octopus	2.551	0.586	4.352	<0.0001	****

Supplementary Table 22. Generalized linear mixed effects model (GLMM) and Tukey Honest Significant Differences (Tukey HSD) pairwise comparisons assessing significant differences in being anchored at phenotype-level and individual-level, depending on the presence of blue goatfish (BG, 2 levels: YES or NO). Frequencies of being anchored (i.e. failing an initiation attempt) were calculated within a finite time interval (1.66 minutes, i.e. the duration of one block) and the number of individuals present in that block, minus the puller ($\boldsymbol{n-1}$).Scene (hunting group),individual identity (individual),and block (hunting subgroup) were used as a nested random effect, with the formula: *anchored ~ phenotype * BG + (1| scene/individual/block) + offset(log(1.66**(n-1)*)), family = poisson*. Presented are coefficient estimates, standard errors (SE), standard deviation (Std. Dev.), z-values, and p-values (Tukey-adjusted), with statistical significance shown as '****' for $p < 0.0001$, '***' for $p < 0.001$, '**' for $p < 0.01$, and '*' for $p < 0.05$.

Random effects

Groups	*Name*	*Variance*	*Std.Dev.*
block:individual:scene	(Intercept)	0.996	0.998
individual:scene	(Intercept)	0.000	0.000
scene	(Intercept)	0.000	0.000

Number of obs: 334, groups: block:individual:scene, 334; individual:scene, 46; scene, 13

Tukey HSD Pairwise comparisons

YES-NO contrasts for phenotypes	*Estimate*	*SE*	*z-value*	*p-value*	
blacktip	0.176	0.096	1.843	0.0653	
barbel goatfish	2.337	0.456	5.122	<0.0001	****
octopus	0.731	0.185	3.949	<0.0001	****

BG contrast for group	*Estimate*	*SE*	*z-value*	*p-value*	
YES - NO	0.761	0.119	6.406	<0.0001	****

Pairwise contrasts (BG = No)	*Estimate*	*SE*	*z-value*	*p-value*	
blacktip - barbel goatfish	-2.545	0.429	-5.926	<0.0001	****
blacktip - octopus	-0.417	0.188	-2.215	0.0686	
barbel goatfish - octopus	2.128	0.456	4.665	<0.0001	****

Supplementary Table 23. Generalized linear mixed effects model (GLMM) and Tukey Honest Significant Differences (Tukey HSD) pairwise comparisons assessing significant differences in pulling efficiency at phenotype-level and individual-level, depending on the presence of blue goatfish (BG, 2 levels: YES or NO). Efficiencies in pulling others were calculated as the frequency of pulling weighted by the frequency of initiations. An autocorrelation structure based on incremental 'blocks' was successfully fitted (Corr), with scene (hunting group) and identity (individual) used as a nested random effect, in the formula: *pulling efficiency ~ phenotype * BG + ar1(block + 0|scene/individual), family = binomial*. Presented are coefficient estimates, standard errors (SE), standard deviation (Std. Dev.), z-values, and p-values (Tukey-adjusted), with statistical significance shown as '****' for $p < 0.0001$, '***' for $p < 0.001$, '**' for $p < 0.01$, and '*' for $p < 0.05$.

Random effects

Groups	*Name*	*Variance*	*Std.Dev.*	*Corr*
individual:scene	block1	0.698	0.836	0.85 (ar1)
scene	block1	0.889	0.943	0.73 (ar1)

Number of obs: 334, groups: block:individual:scene, 334; individual:scene, 46; scene, 13

Tukey HSD Pairwise comparisons

YES-NO contrasts for phenotypes	*Estimate*	*SE*	*z-value*	*p-value*	
blacktip	-0.181	0.181	-1.005	0.3149	
barbel goatfish	-0.255	0.158	-1.613	0.1067	
octopus	-0.349	0.140	-2.500	0.0124	*

BG contrast for group	*Estimate*	*SE*	*z-value*	*p-value*	
YES - NO	-0.303	0.115	-2.642	0.0082	**

Pairwise contrasts (BG = No)	*Estimate*	*SE*	*z-value*	*p-value*
blacktip - barbel goatfish	0.235	0.113	2.082	0.0936
blacktip - octopus	-0.032	0.153	-0.210	0.9761
barbel goatfish - octopus	-0.267	0.132	-2.028	0.1055

Supplementary Table 24. Generalized linear mixed effects model (GLMM) assessing significant differences in mean distance to centroid depending on the presence of blue goatfish (BG, 2 levels: YES or NO). Distance to centroid was calculated as the mean distance to the group's centroid across the block (i.e. 1.66 minutes) of all individuals ($\boldsymbol{n}$). An autocorrelation structure based on incremental 'blocks' was successfully fitted (Corr), with scene (hunting group) used as a nesting random effect, in the formula: *centroid distance ~ + ar1(block + 0| scene), family =gaussian*. Presented are coefficient estimates, standard errors (SE), standard deviation (Std. Dev.), z-values, and p-values (Tukey-adjusted), with statistical significance shown as '****' for p < 0.0001, '***' for p < 0.001, '**' for p < 0.01, and '*' for p < 0.05.

Random effects

Groups	*Name*	*Variance*	*Std.Dev.*	*Corr*
timeblock:scene	(Intercept)	0.1408	0.3753	0.93 (ar1)
scene	(Intercept)	3.087	1.757	

Number of obs: 107, groups: scene, 13

Conditional model

	Estimate	*SE*	*z-value*	*p-value*	
(Intercept)	-3.338	0.582	-5.739	<0.0001	****
BG YES	-2.074	0.501	-4.138	<0.0001	****

Supplementary Table 25. Generalized linear mixed effects model (GLMM) assessing significant differences in centroid displacement depending on the presence of blue goatfish (BG, 2 levels: YES or NO). Displacement was calculated as the distance between the start and end positions of each block (i.e. distance travelled in 1.66 minutes). Scene (hunting group) and block (subgroup) were used as a nested random effect, in the formula: *centroid displacement ~ bg + (1| scene/block) + offset(n), family = Gamma(link = log)*. Presented are coefficient estimates, standard errors (SE), standard deviation (Std. Dev.), z-values, and p-values (Tukey-adjusted), with statistical significance shown as '****' for $p < 0.0001$, '***' for $p < 0.001$, '**' for $p < 0.01$, and '*' for $p < 0.05$.

Random effects

Groups	*Name*	*Variance*	*Std.Dev.*
block:scene	(Intercept)	0.055	0.234
scene	(Intercept)	0.174	0.417

Number of obs: 107, groups: scene, 13

Conditional model

	Estimate	*SE*	*z-value*	*p-value*	
(Intercept)	1.460	0.172	8.462	<0.0001	****
BG YES	0.078	0.186	0.419	0.6750	

Supplementary Table 26. Generalized linear mixed effects model (GLMM) assessing significant differences in centroid displacement depending on the presence of extreme phenotypes (3 levels: BTG, BG, and BTG&BG). Displacement was calculated as the distance between the start and end positions of each block (i.e. distance travelled in 1.66 minutes). An autocorrelation structure based on incremental 'blocks' was successfully fitted (Corr), with scene (hunting group) used as a nesting random effect, in the formula: *centroid displacement ~ extreme phenotypes + ar1(block + 0| scene), family = Gamma(link = log)*. Presented are coefficient estimates, standard errors (SE), standard deviation (Std. Dev.), z-values, and p-values (Tukey-adjusted), with statistical significance shown as '****' for p < 0.0001, '***' for p < 0.001, '**' for p < 0.01, and '*' for p < 0.05.

Random effects

Groups	*Name*	*Variance*	*Std.Dev.*	*Corr*
scene	block1	0.09004	0.3001	0.99 (ar1)

Number of obs: 107, groups: scene, 13

Conditional model

	Estimate	*SE*	*z-value*	*p-value*	
(Intercept)	1.814	0.131	13.807	<0.0001	***
BTG	-0.606	0.207	-2.922	0.0035	**
BTG&BG	-0.489	0.206	-2.367	0.0179	*

Tukey HSD Pairwise contrasts

	Estimate	*SE*	*z-value*	*p-value*	
BG - BTG	2.786	0.964	2.891	0.0107	*
BG - BTG&BG	2.371	0.962	2.464	0.0366	*
BTG - BTG&BG	-0.415	0.907	-0.458	0.891	

Supplementary Table 27. Generalized linear mixed effects model (GLMM) assessing significant differences in mean distance to centroid depending on the presence of extreme phenotypes (3 levels: BTG, BG, and BTG&BG). Distance to centroid was calculated as the mean distance to the group's centroid across the block (i.e. 1.66 minutes) of all individuals ($\boldsymbol{n}$). Scene (hunting group) and block (subgroup) were used as a nested random effect, in the formula: *centroid distance ~ extreme phenotypes + (1| scene/block) + offset(n), family = Gamma(link = log)*. Presented are coefficient estimates, standard errors (SE), standard deviation (Std. Dev.), z-values, and p-values (Tukey-adjusted), with statistical significance shown as '****' for p < 0.0001, '***' for p < 0.001, '**' for p < 0.01, and '*' for p < 0.05.

Random effects

Groups	***Name***	***Variance***	***Std.Dev.***
timeblock:scene	(Intercept)	0.253	0.503
scene	(Intercept)	0.124	0.353

Number of obs: 99, groups: block:scene, 99; scene, 12

Conditional model

	Estimate	***SE***	***z-value***	***p-value***	
(Intercept)	-2.090	0.161	-13.020	<0.0001	***
BTG	-0.670	0.256	-2.612	0.0090	**
BTG&BG	-0.538	0.234	-2.302	0.0213	*

Tukey HSD Pairwise contrasts

	Estimate	***SE***	***z-value***	***p-value***	
BG - BTG	0.060	0.024	2.568	0.0276	*
BG - BTG&BG	0.052	0.022	2.355	0.0486	*
BTG - BTG&BG	-0.009	0.020	-0.438	0.8997	

Supplementary Table 28. Generalized linear mixed effects model (GLMM) assessing significant differences in the outcome of initiations (2 levels: successful, i.e. pull, and unsuccessful, i.e. anchor) depending on kinematic descriptors of the initiation attempt (between $t1$ and $t2$): speed, distance to centroid, tortuosity, and initiator-follower angle. Data were standardized by scaling using the median and interquartile range. Scene (hunting group), species identity (species) and individual identity (individual) were used as a nested random effect, in the formula: *initiation outcome ~ speed + centroid distance + tortuosity + initiator-follower angle + (1| scene/species/individual), family = binomial)*. Presented are coefficient estimates, standard errors (SE), standard deviation (Std. Dev.), z-values, and p-values (Tukey-adjusted), with statistical significance shown as '****' for p < 0.0001, '***' for p < 0.001, '**' for p < 0.01, and '*' for p < 0.05.

Random effects

Groups	***Name***	***Variance***	***Std.Dev.***
individual:species:scene	(Intercept)	0.595	0.771
species:scene	(Intercept)	0.262	0.511
scene	(Intercept)	0.000	0.000

Number of obs: 1180, groups: individual:species:scene, 69; species:scene, 44; scene, 13

Conditional model

	**Estimate**	**SE**	**z-value**	**p-value**	
(Intercept)	0.672	0.220	3.058	0.0022	**
Speed	0.211	0.084	2.503	0.0123	*
Distance to centroid	-0.210	0.080	-2.628	0.0086	**
Tortuosity	-0.682	0.139	-4.918	<0.0001	****
Initiator-follower angle	-0.500	0.083	-6.056	<0.0001	****

Supplementary Table 29. Generalized linear mixed effects model (GLMM) assessing significant differences in average individual speed between phenotypes. Scene (hunting group), species identity (species) and individual identity (individual) were used as a nested random effect, in the formula: *speed ~ phenotype + (1| scene/species/individual), family =Gamma(link=log)).* Presented are coefficient estimates, standard errors (SE), standard deviation (Std. Dev.), z-values, and p-values (Tukey-adjusted), with statistical significance shown as '****' for $p < 0.0001$, '***' for $p < 0.001$, '**' for $p < 0.01$, and '*' for $p < 0.05$.

Random effects

Groups	*Name*	*Variance*	*Std.Dev.*
block:individual:scene	(Intercept)	0.000	0.000
individual:scene	(Intercept)	0.024	0.153
scene	(Intercept)	0.118	0.344

Number of obs: 501, groups: block:individual:scene, 501; individual:scene, 75; scene, 13

Conditional model

	Estimate	*SE*	*z-value*	*p-value*	
(Intercept)	-2.241	0.122	-18.308	<0.0001	****
barbel goatfish	0.236	0.100	2.368	0.0179	*
lyretail	0.468	0.142	3.304	0.0010	**
octopus	0.110	0.101	1.090	0.2757	
blue goatfish	0.699	0.114	6.128	<0.0001	****
yellow goatfish	0.900	0.163	5.519	<0.0001	****

Tukey HSD Pairwise comparisons

Contrast	*Estimate*	*SE*	*z-value*	*p-value*	
blacktip - barbel goatfish	-0.028	0.013	-2.252	0.2143	
blacktip - lyretail	-0.063	0.022	-2.829	0.0530	
blacktip - octopus	-0.012	0.011	-1.085	0.8876	
blacktip - blue goatfish	-0.108	0.022	-4.972	<0.0001	****
blacktip - yellow goatfish	-0.155	0.041	-3.771	0.0023	**
barbel goatfish - lyretail	-0.035	0.023	-1.541	0.6378	
barbel goatfish - octopus	0.016	0.014	1.148	0.8612	
barbel goatfish - blue goatfish	-0.079	0.022	-3.662	0.0034	**
barbel goatfish - yellow goatfish	-0.127	0.041	-3.073	0.0258	*
lyretail - octopus	0.051	0.022	2.355	0.1723	
lyretail - blue goatfish	-0.044	0.024	-1.851	0.4327	*
lyretail - yellow goatfish	-0.092	0.043	-2.160	0.2567	
octopus - blue goatfish	-0.095	0.020	-4.695	<0.0001	****
octopus - yellow goatfish	-0.143	0.041	-3.509	0.0060	**
blue goatfish - yellow goatfish	-0.048	0.040	-1.206	0.8340	

Supplementary Table 30. Generalized linear mixed effects model (GLMM) assessing significant differences in average distance to centroid between phenotypes. Scene (hunting group), species identity (species) and individual identity (individual) were used as a nested random effect, in the formula: *centroid distance ~ phenotype + (1| scene/species/individual), family =Gamma(link=log))*. Presented are coefficient estimates, standard errors (SE), standard deviation (Std. Dev.), z-values, and p-values (Tukey-adjusted), with statistical significance shown as '****' for $p < 0.0001$, '***' for $p < 0.001$, '**' for $p < 0.01$, and '*' for $p < 0.05$.

Random effects

Groups	*Name*	*Variance*	*Std.Dev.*
block:individual:scene	(Intercept)	0.339	0.582
individual:scene	(Intercept)	0.007	0.086
scene	(Intercept)	0.299	0.547

Number of obs: 501, groups: block:individual:scene, 501; individual:scene, 75; scene, 13

Conditional model

	Estimate	*SE*	*z-value*	*p-value*	
(Intercept)	-0.672	0.169	-3.976	0.0001	***
barbel goatfish	-0.435	0.089	-4.859	<0.0001	****
lyretail	-0.022	0.139	-0.158	0.8743	
octopus	-0.464	0.094	-4.932	<0.0001	****
blue goatfish	-0.208	0.110	-1.899	0.0576	
yellow goatfish	-0.028	0.163	-0.170	0.8650	

Tukey HSD Pairwise comparisons

Contrast	*Estimate*	*SE*	*z-value*	*p-value*	
blacktip - barbel goatfish	0.011	0.070	0.159	1.0000	
blacktip - lyretail	0.190	0.051	3.755	0.0024	**
blacktip - octopus	0.096	0.053	1.810	0.4592	
blacktip - blue goatfish	0.014	0.081	0.171	1.0000	
blacktip - yellow goatfish	-0.169	0.067	-2.507	0.1219	
barbel goatfish - lyretail	0.010	0.032	0.307	0.9996	
barbel goatfish - octopus	-0.084	0.043	-1.956	0.3682	
barbel goatfish - blue goatfish	-0.166	0.080	-2.068	0.3044	
barbel goatfish - yellow goatfish	0.179	0.065	2.756	0.0648	
lyretail - octopus	0.085	0.060	1.408	0.7223	
lyretail - blue goatfish	0.003	0.087	0.033	1.0000	
lyretail - yellow goatfish	-0.094	0.039	-2.426	0.1473	
octopus - blue goatfish	-0.176	0.078	-2.266	0.2080	
octopus - yellow goatfish	-0.082	0.074	-1.111	0.8769	
blue goatfish - yellow goatfish	0.011	0.070	0.159	1.0000	

Supplementary Table 31. Generalized linear mixed effects model (GLMM) assessing significant differences in average movement tortuosity between phenotypes. Scene (hunting group), species identity (species) and individual identity (individual) were used as a nested random effect, in the formula: *tortuosity ~ phenotype + (1| scene/species/individual), family =Gamma(link=log)).* Presented are coefficient estimates, standard errors (SE), standard deviation (Std. Dev.), z-values, and p-values (Tukey-adjusted), with statistical significance shown as '****' for p < 0.0001, '***' for p < 0.001, '**' for p < 0.01, and '*' for p < 0.05.

Random effects

Groups	*Name*	*Variance*	*Std.Dev.*
block:individual:scene	(Intercept)	0.002	0.048
individual:scene	(Intercept)	0.003	0.053
scene	(Intercept)	0.008	0.092

Number of obs: 501, groups: block:individual:scene, 501; individual:scene, 75; scene, 13

Conditional model

	Estimate	*SE*	*z-value*	*p-value*	
(Intercept)	0.222	0.033	6.798	<0.0001	****
barbel goatfish	0.036	0.026	1.374	0.1690	
lyretail	0.027	0.037	0.739	0.4600	
octopus	0.032	0.027	1.166	0.2440	
blue goatfish	0.024	0.031	0.799	0.4250	
yellow goatfish	-0.019	0.041	-0.452	0.6510	

Tukey HSD Pairwise comparisons

Contrast	*Estimate*	*SE*	*z-value*	*p-value*
blacktip - barbel goatfish	-0.036	0.026	-1.374	0.7425
blacktip - lyretail	-0.027	0.037	-0.739	0.9769
blacktip - octopus	-0.032	0.027	-1.166	0.8530
blacktip - blue goatfish	-0.024	0.031	-0.799	0.9677
blacktip - yellow goatfish	0.019	0.041	0.452	0.9976
barbel goatfish - lyretail	0.009	0.037	0.240	0.9999
barbel goatfish - octopus	0.004	0.029	0.157	1.0000
barbel goatfish - blue goatfish	0.012	0.031	0.383	0.9989
barbel goatfish - yellow goatfish	0.055	0.043	1.288	0.7916
lyretail - octopus	-0.004	0.034	-0.128	1.0000
lyretail - blue goatfish	0.003	0.033	0.090	1.0000
lyretail - yellow goatfish	0.046	0.045	1.018	0.9117
octopus - blue goatfish	0.007	0.027	0.273	0.9998
octopus - yellow goatfish	0.050	0.040	1.257	0.8083
blue goatfish - yellow goatfish	0.043	0.039	1.104	0.8798

Supplementary Table 32. Generalized linear mixed effects model (GLMM) assessing significant differences in average individual angle in relation to the centroid's movement vector, between phenotypes. Scene (hunting group), species identity (species) and individual identity (individual) were used as a nested random effect, in the formula: *centroid angle ~ phenotype + (1| scene/species/individual), family = gaussian).* Presented are coefficient estimates, standard errors (SE), standard deviation (Std. Dev.), z-values, and p-values (Tukey-adjusted), with statistical significance shown as '****' for $p < 0.0001$, '***' for $p < 0.001$, '**' for $p < 0.01$, and '*' for $p < 0.05$.

Random effects

Groups	*Name*	*Variance*	*Std.Dev.*
block:individual:scene	(Intercept)	193.919	13.925
individual:scene	(Intercept)	41.425	6.436
scene	(Intercept)	15.809	3.976

Number of obs: 501, groups: block:individual:scene, 501; individual:scene, 75; scene, 13

Conditional model

	Estimate	*SE*	*z-value*	*p-value*	
(Intercept)	55.917	2.508	22.295	<0.0001	****
barbel goatfish	-8.236	3.262	-2.525	0.0116	*
lyretail	-9.843	4.304	-2.287	0.0222	*
octopus	-9.267	3.227	-2.872	0.0041	**
blue goatfish	-11.702	3.45	-3.392	0.0007	***
yellow goatfish	-8.485	5.134	-1.653	0.0984	

Tukey HSD Pairwise comparisons

Contrast	*Estimate*	*SE*	*z-value*	*p-value*	
blacktip - barbel goatfish	8.236	3.260	2.525	0.1188	
blacktip - lyretail	9.843	4.300	2.287	0.2013	
blacktip - octopus	9.267	3.230	2.872	0.0485	*
blacktip - blue goatfish	11.702	3.450	3.392	0.0097	**
blacktip - yellow goatfish	8.485	5.130	1.653	0.5639	
barbel goatfish - lyretail	1.607	4.490	0.358	0.9992	
barbel goatfish - octopus	1.031	3.530	0.292	0.9997	
barbel goatfish - blue goatfish	3.466	3.690	0.939	0.9362	
barbel goatfish - yellow goatfish	0.249	5.380	0.046	1.0000	
lyretail - octopus	-0.576	4.250	-0.135	1.0000	
lyretail - blue goatfish	1.859	4.090	0.454	0.9976	
lyretail - yellow goatfish	-1.358	5.650	-0.240	0.9999	
octopus - blue goatfish	2.435	3.330	0.732	0.9779	
octopus - yellow goatfish	-0.782	5.090	-0.154	1.0000	
blue goatfish - yellow goatfish	-3.217	4.890	-0.657	0.9864	

Supplementary Table 33. Generalized linear mixed effects model (GLMM) assessing significant differences in average individual speed during initiations, between phenotypes. Scene (hunting group) and individual identity (individual) were used as a nested random effect, in the formula: *speed ~ phenotype + (1| scene/ individual), family =Gamma(link=log))*. Presented are coefficient estimates, standard errors (SE), standard deviation (Std. Dev.), z-values, and p-values (Tukey-adjusted), with statistical significance shown as '****' for p < 0.0001, '***' for p < 0.001, '**' for p < 0.01, and '*' for p < 0.05.

Random effects

Groups	***Name***	***Variance***	***Std.Dev.***
individual:scene	(Intercept)	0.080	0.284
scene	(Intercept)	0.151	0.389

Number of obs: 1180, groups: block:individual:scene, 1180; individual:scene, 69; scene, 13

Conditional model

	Estimate	***SE***	***z-value***	***p-value***	
(Intercept)	-1.829	0.152	-12.001	<0.0001	****
barbel goatfish	-0.109	0.135	-0.809	0.4180	
lyretail	0.235	0.195	1.204	0.2290	
octopus	0.218	0.152	1.438	0.1500	
blue goatfish	0.609	0.155	3.926	<0.0001	****
yellow goatfish	0.818	0.201	4.068	<0.0001	****

Tukey HSD Pairwise comparisons

Contrast	***Estimate***	***SE***	***z-value***	***p-value***	
blacktip - barbel goatfish	0.017	0.021	0.803	0.9670	
blacktip - lyretail	-0.042	0.037	-1.136	0.8663	
blacktip - octopus	-0.039	0.028	-1.397	0.7289	
blacktip - blue goatfish	-0.135	0.039	-3.493	0.0063	**
blacktip - yellow goatfish	-0.203	0.068	-3.006	0.0317	*
barbel goatfish - lyretail	-0.059	0.037	-1.620	0.5852	
barbel goatfish - octopus	-0.056	0.028	-2.022	0.3298	
barbel goatfish - blue goatfish	-0.151	0.038	-3.970	0.0010	**
barbel goatfish - yellow goatfish	-0.220	0.069	-3.204	0.0170	*
lyretail - octopus	0.003	0.039	0.086	1.0000	
lyretail - blue goatfish	-0.092	0.040	-2.282	0.2012	
lyretail - yellow goatfish	-0.161	0.070	-2.298	0.1949	
octopus - blue goatfish	-0.095	0.038	-2.512	0.1204	
octopus - yellow goatfish	-0.164	0.068	-2.419	0.1495	
blue goatfish - yellow goatfish	-0.069	0.064	-1.074	0.8920	

Supplementary Table 34. Generalized linear mixed effects model (GLMM) assessing significant differences in average distance to centroid during initiations, between phenotypes. Scene (hunting group) and individual identity (individual) were used as a nested random effect, in the formula: *centroid distance ~ phenotype + (1| scene/ individual), family =Gamma(link=log))*. Presented are coefficient estimates, standard errors (SE), standard deviation (Std. Dev.), z-values, and p-values (Tukey-adjusted), with statistical significance shown as '****' for $p < 0.0001$, '***' for $p < 0.001$, '**' for $p < 0.01$, and '*' for $p < 0.05$.

Random effects

Groups	*Name*	*Variance*	*Std.Dev.*
individual:scene	(Intercept)	0.069	0.262
scene	(Intercept)	0.270	0.519

Number of obs: 1180, groups: block:individual:scene, 1180; individual:scene, 69; scene, 13

Conditional model

	Estimate	*SE*	*z-value*	*p-value*	
(Intercept)	-0.818	0.181	-4.529	<0.0001	****
barbel goatfish	-0.248	0.133	-1.859	0.0630	
lyretail	0.153	0.198	0.772	0.4400	
octopus	-0.169	0.152	-1.116	0.2640	
blue goatfish	-0.021	0.156	-0.136	0.8920	
yellow goatfish	0.116	0.200	0.582	0.5610	

Tukey HSD Pairwise comparisons

Contrast	*Estimate*	*SE*	*z-value*	*p-value*
blacktip - barbel goatfish	0.097	0.055	1.758	0.4931
blacktip - lyretail	-0.073	0.098	-0.745	0.9763
blacktip - octopus	0.069	0.063	1.100	0.8814
blacktip - blue goatfish	0.009	0.068	0.136	1.0000
blacktip - yellow goatfish	-0.054	0.096	-0.565	0.9932
barbel goatfish - lyretail	-0.170	0.094	-1.801	0.4652
barbel goatfish - octopus	-0.028	0.055	-0.511	0.9958
barbel goatfish - blue goatfish	-0.088	0.060	-1.460	0.6900
barbel goatfish - yellow goatfish	-0.151	0.095	-1.589	0.6056
lyretail - octopus	0.142	0.093	1.527	0.6467
lyretail - blue goatfish	0.082	0.085	0.963	0.9297
lyretail - yellow goatfish	0.019	0.113	0.164	1.0000
octopus - blue goatfish	-0.060	0.059	-1.011	0.9144
octopus - yellow goatfish	-0.123	0.094	-1.313	0.7780
blue goatfish - yellow goatfish	-0.064	0.086	-0.736	0.9775

Supplementary Table 35. Generalized linear mixed effects model (GLMM) assessing significant differences in average movement tortuosity during initiations, between phenotypes. Scene (hunting group) and individual identity (individual) were used as a nested random effect, in the formula: *tortuosity ~ phenotype + (1| scene/ individual), family = gaussian).* Presented are coefficient estimates, standard errors (SE), standard deviation (Std. Dev.), z-values, and p-values (Tukey-adjusted), with statistical significance shown as '****' for $p < 0.0001$, '***' for $p < 0.001$, '**' for $p < 0.01$, and '*' for $p < 0.05$.

Random effects

Groups	*Name*	*Variance*	*Std.Dev.*
individual:scene	(Intercept)	0.002	0.041
scene	(Intercept)	0.011	0.107

Number of obs: 1180, groups: block:individual:scene, 1180; individual:scene, 69; scene, 13

Conditional model

	Estimate	*SE*	*z-value*	*p-value*	
(Intercept)	0.150	0.036	4.135	<0.0001	****
barbel goatfish	0.089	0.023	3.797	0.0001	***
lyretail	0.041	0.036	1.138	0.2552	
octopus	0.045	0.028	1.617	0.1058	
blue goatfish	0.065	0.028	2.330	0.0198	*
yellow goatfish	0.048	0.035	1.370	0.1707	

Tukey HSD Pairwise comparisons

Contrast	*Estimate*	*SE*	*z-value*	*p-value*	
blacktip - barbel goatfish	-0.089	0.023	-3.797	0.0021	**
blacktip - lyretail	-0.041	0.036	-1.138	0.8656	
blacktip - octopus	-0.045	0.028	-1.617	0.5872	
blacktip - blue goatfish	-0.065	0.028	-2.330	0.1830	
blacktip - yellow goatfish	-0.048	0.035	-1.370	0.7450	
barbel goatfish - lyretail	0.048	0.035	1.373	0.7433	
barbel goatfish - octopus	0.044	0.027	1.636	0.5750	
barbel goatfish - blue goatfish	0.024	0.026	0.902	0.9460	
barbel goatfish - yellow goatfish	0.041	0.035	1.168	0.8519	
lyretail - octopus	-0.004	0.035	-0.106	1.0000	
lyretail - blue goatfish	-0.024	0.031	-0.780	0.9709	
lyretail - yellow goatfish	-0.007	0.040	-0.171	1.0000	
octopus - blue goatfish	-0.020	0.027	-0.766	0.9732	
octopus - yellow goatfish	-0.003	0.036	-0.088	1.0000	
blue goatfish - yellow goatfish	0.017	0.031	0.559	0.9935	

Supplementary Table 36. Generalized linear mixed effects model (GLMM) assessing significant differences in average angle between initiator and potential follower during initiations, between phenotypes. Scene (hunting group) and individual identity (individual) were used as a nested random effect, in the formula: *initiator angle ~ phenotype + (1| scene/ individual), family = gaussian).* Presented are coefficient estimates, standard errors (SE), standard deviation (Std. Dev.), z-values, and p-values (Tukey-adjusted), with statistical significance shown as '****' for p < 0.0001, '***' for p < 0.001, '**' for p < 0.01, and '*' for p < 0.05.

Random effects

Groups	*Name*	*Variance*	*Std.Dev.*
individual:scene	(Intercept)	12.080	3.476
scene	(Intercept)	23.010	4.797

Number of obs: 1180, groups: block:individual:scene, 1180; individual:scene, 69; scene, 13

Conditional model

	Estimate	*SE*	*z-value*	*p-value*	
(Intercept)	80.561	2.998	26.869	<0.0001	****
barbel goatfish	0.913	3.013	0.303	0.7619	
lyretail	-1.709	4.957	-0.345	0.7303	
octopus	-7.374	3.835	-1.923	0.0545	
blue goatfish	-3.722	3.460	-1.076	0.2821	
yellow goatfish	-4.496	4.377	-1.027	0.3043	

Tukey HSD Pairwise comparisons

Contrast	*Estimate*	*SE*	*z-value*	*p-value*
blacktip - barbel goatfish	-0.913	3.01	-0.303	0.9997
blacktip - lyretail	1.709	4.960	0.345	0.9994
blacktip - octopus	7.374	3.840	1.923	0.3888
blacktip - blue goatfish	3.722	3.460	1.076	0.8911
blacktip - yellow goatfish	4.495	4.380	1.027	0.9089
barbel goatfish - lyretail	2.621	4.670	0.561	0.9934
barbel goatfish - octopus	8.286	3.560	2.328	0.1835
barbel goatfish - blue goatfish	4.635	3.080	1.504	0.6621
barbel goatfish - yellow goatfish	5.408	4.210	1.283	0.7942
lyretail - octopus	5.665	4.930	1.149	0.8604
lyretail - blue goatfish	2.013	4.170	0.483	0.9968
lyretail - yellow goatfish	2.787	5.340	0.522	0.9953
octopus - blue goatfish	-3.652	3.550	-1.028	0.9085
octopus - yellow goatfish	-2.878	4.550	-0.632	0.9886
blue goatfish - yellow goatfish	0.773	3.830	0.202	1.0000

Supplementary Table 37. 2-sample tests for equality of proportions with continuity correction assessing significant differences between phenotypes in displacements, both as actors (Act. Displ.) and receivers (Rec. Displ.), in proportion to the total number of individuals of said phenotype present in groups (n). Statistical significance shown as '****' for $p < 0.0001$, '***' for $p < 0.001$, '**' for $p < 0.01$, and '*' for $p < 0.05$.

Comparisons	*Act. Displ.*	*n*	χ^2	*p-value*	
octopus vs. blacktip	(39,0)	(107,132)	54.856	<0.0001	****
octopus vs. barbel goatfish	(39,3)	(107,95)	31.873	<0.0001	****
octopus vs. lyretail	(39,6)	(107,42)	6.016	0.0142	*
octopus vs. blue goatfish	(39,2)	(107,102)	37.232	<0.0001	****
octopus vs. yellow goatfish	(39,0)	(107,23)	10.303	0.0013	**
blacktip vs. barbel goatfish	(0,3)	(132,95)	2.150	0.1426	****
blacktip vs. lyretail	(0,6)	(132,42)	15.475	<0.0001	****
blacktip vs. blue goatfish	(0,2)	(132,102)	0.809	0.3683	
blacktip vs. yellow goatfish	(0,0)	(132,23)	-	-	
barbel goatfish vs. lyretail	(3,6)	(95,42)	4.203	0.0404	*
barbel goatfish vs. blue goatfish	(3,2)	(95,102)	0.006	0.9358	
barbel goatfish vs. yellow goatfish	(3,0)	(95,23)	0.016	0.9004	
lyretail vs. blue goatfish	(6,2)	(42,102)	6.424	0.0113	*
lyretail vs. yellow goatfish	(6,0)	(42,23)	2.116	0.1458	
blue goatfish vs. yellow goatfish	(2,0)	(102,23)	0.000	1.0000	

Comparisons	*Rec. Displ.*	*n*	χ^2	*p-value*	
octopus vs. blacktip	(0,32)	(107,132)	27.895	<0.0001	****
octopus vs. barbel goatfish	(0,7)	(107,95)	6.113	0.0134	**
octopus vs. lyretail	(0,3)	(107,42)	4.600	0.032	*
octopus vs. blue goatfish	(0,7)	(107,102)	5.626	0.0177	*
octopus vs. yellow goatfish	(0,1)	(107,23)	0.722	0.3954	
blacktip vs. barbel goatfish	(32,7)	(132,95)	9.900	0.0017	**
blacktip vs. lyretail	(32,3)	(132,41)	4.782	0.0288	*
blacktip vs. blue goatfish	(32,7)	(132,102)	11.293	0.0008	***
blacktip vs. yellow goatfish	(32,1)	(132,23)	3.515	0.0608	
barbel goatfish vs. lyretail	(7,3)	(95,42)	0.000	1.0000	
barbel goatfish vs. blue goatfish	(7,7)	(95,102)	0.000	1.0000	
barbel goatfish vs. yellow goatfish	(7,1)	(95,23)	0.003	0.9563	
lyretail vs. blue goatfish	(3,7)	(42,102)	0.000	1.0000	
lyretail vs. yellow goatfish	(3,1)	(42,23)	0.000	1.0000	
blue goatfish vs. yellow goatfish	(7,1)	(102,23)	0.000	1.0000	

Supplementary Table 38. 2-sample tests for equality of proportions with continuity correction assessing significant differences between phenotypes as targets of punching, in proportion to the blocks where said phenotype was present in subgroups (n). Statistical significance shown as '****' for p < 0.0001, '***' for p < 0.001, '**' for p < 0.01, and '*' for p < 0.05.

Comparisons	***punches***	***n***	χ^2	***p-value***	
blacktip vs. barbel goatfish	(27,6)	(60,80)	20.151	<0.0001	****
blacktip vs. lyretail	(27,3)	(60,36)	9.959	0.0016	**
blacktip vs. blue goatfish	(27,3)	(60,58)	21.259	<0.0001	****
blacktip vs. yellow goatfish	(27,0)	(60,18)	10.087	0.0015	**
barbel goatfish vs. lyretail	(6,3)	(80,36)	0.000	1.0000	
barbel goatfish vs. blue goatfish	(6,3)	(80,58)	0.036	0.8492	
barbel goatfish vs. yellow goatfish	(6,0)	(80,18)	0.442	0.5060	
lyretail vs. blue goatfish	(3,3)	(36,58)	0.091	0.7627	
lyretail vs. yellow goatfish	(3,0)	(36,18)	0.518	0.4718	
blue goatfish vs. yellow goatfish	(3,0)	(58,18)	0.092	0.7615	

Supplementary Table 39. Generalized linear mixed effects model (GLMM) assessing significant differences in probability of punching occurring depending on presence or absence of blacktips (BTG) in subgroups. Scene (hunting group) and block (subgroup) were used as a nested random effect, in the formula: *punch ~ btg + (1| scene/block), family = binomial*. Presented are coefficient estimates, standard errors (SE), standard deviation (Std. Dev.), z-values, and p-values (Tukey-adjusted), with statistical significance shown as '****' for p < 0.0001, '***' for p < 0.001, '**' for p < 0.01, and '*' for p < 0.05.

Random effects

Groups	***Name***	***Variance***	***Std.Dev.***
block:scene	(Intercept)	0.000	0.000
scene	(Intercept)	0.034	0.185

Number of obs: 107, groups: block:scene, 107; scene, 13

Conditional model

	Estimate	***SE***	***z-value***	***p-value***	
(Intercept)	-2.693	0.606	-4.448	<0.0001	****
BTG = Yes	2.287	0.663	3.449	0.0006	***

Supplementary Table 40. Generalized linear mixed effects model (GLMM) assessing significant differences in probability of punching occurring depending on presence or absence of blue goatfish (BG) in subgroups. Scene (hunting group) and block (subgroup) were used as a nested random effect, in the formula: *punch ~ bg + (1| scene/block), family = binomial*. Presented are coefficient estimates, standard errors (SE), standard deviation (Std. Dev.), z-values, and p-values (Tukey-adjusted), with statistical significance shown as '****' for p < 0.0001, '***' for p < 0.001, '**' for p < 0.01, and '*' for p < 0.05.

Random effects

Groups	*Name*	*Variance*	*Std.Dev.*
block:scene	(Intercept)	0.000	0.012
scene	(Intercept)	0.000	0.000

Number of obs: 107, groups: block:scene, 107; scene, 13

Conditional model

	Estimate	*SE*	*z-value*	*p-value*	
(Intercept)	-0.288	0.294	-0.979	0.3275	
BG = Yes	-1.872	0.576	-3.253	0.0011	**

Supplementary Table 41. Generalized linear mixed effects model (GLMM) assessing significant differences in probability of punching occurring depending on the presence of extreme phenotypes. Scene (hunting group) and block (subgroup) were used as a nested random effect, in the formula: *punch ~ extreme phenotypes + (1| scene/block), family = binomial*. Presented are coefficient estimates, standard errors (SE), standard deviation (Std. Dev.), z-values, and p-values (Tukey-adjusted), with statistical significance shown as '****' for $p < 0.0001$, '***' for $p < 0.001$, '**' for $p < 0.01$, and '*' for $p < 0.05$.

Random effects

Groups	*Name*	*Variance*	*Std.Dev.*
block:scene	(Intercept)	0.000	0.011
scene	(Intercept)	0.000	0.000

Number of obs: 99, groups: block:scene, 99; scene, 12

Conditional model

	Estimate	*SE*	*z-value*	*p-value*	
(Intercept)	-2.9178	0.7945	-3.6730	0.0002	***
BTG	2.8690	0.8504	3.3740	0.0007	***
BTG&BG	1.5960	0.9256	1.7240	0.0847	

Tukey HSD Pairwise contrasts

	Estimate	*SE*	*z-value*	*p-value*	
BG - BTG	-0.437	0.087	-5.032	<0.0001	****
BG - BTG&BG	-0.159	0.102	-1.565	0.2609	
BTG - BTG&BG	0.277	0.126	2.199	0.0712	

Supplementary Table 42. Generalized linear mixed effects model (GLMM) assessing significant differences in probability of punching occurring depending on subgroup displacement. Scene (hunting group) and block (subgroup) were used as a nested random effect, in the formula: *punch ~ disp_cent + (1| scene/block), family = binomial*. Presented are coefficient estimates, standard errors (SE), standard deviation (Std. Dev.), z-values, and p-values (Tukey-adjusted), with statistical significance shown as '****' for $p < 0.0001$, '***' for $p < 0.001$, '**' for $p < 0.01$, and '*' for $p < 0.05$.

Random effects

Groups	***Name***	***Variance***	***Std.Dev.***
block:scene	(Intercept)	0.000	0.000
scene	(Intercept)	1.177	1.085

Number of obs: 107, groups: block:scene, 107; scene, 13

Conditional model

	Estimate	***SE***	***z-value***	***p-value***	
(Intercept)	0.501	0.794	0.631	0.5282	
Displacement	-0.439	0.181	-2.424	0.0153	*

Supplementary Table 43. Generalized linear mixed effects model (GLMM) assessing significant differences in the likelihood of performing a web-over on structures (yes or no), depending on their status (full or empty) and occurrence or not of fish attack (and therefore presence of fish). Individual identity (individual) was used as a random effect, in the formula: *yes/no web-over ~ status * fish attack + (1| individual), family = binomial*. Presented are coefficient estimates, standard errors (SE), standard deviation (Std. Dev.), z-values, and p-values (Tukey-adjusted), with statistical significance shown as '****' for $p < 0.0001$, '***' for $p < 0.001$, '**' for $p < 0.01$, and '*' for $p < 0.05$.

Random effects

Groups	*Name*	*Variance*	*Std.Dev.*
individual	(Intercept)	1.024	1.012

Number of obs: 113, groups: individual, 48

Conditional model

	Estimate	*SE*	*z-value*	*p-value*	
(Intercept)	0.0324	0.4101	0.0790	0.9371	
Fish attack	2.9722	1.0334	2.8760	0.0040	**
Status empty	-0.5226	0.5488	-0.9520	0.3410	
Fish attack : Status	-1.8728	1.2063	-1.5520	0.1206	

Supplementary Table 44. Generalized linear mixed effects model (GLMM) assessing significant differences in time spent in web-over probing structures, depending on their status (full or empty) and occurrence or not of fish attack (and therefore presence of fish). Individual identity (individual) was used as a random effect, in the formula: *time ~ status * fish attack + (1| individual), family = gaussian*. Presented are coefficient estimates, standard errors (SE), standard deviation (Std. Dev.), z-values, and p-values (Tukey-adjusted), with statistical significance shown as '****' for $p < 0.0001$, '***' for $p < 0.001$, '**' for $p < 0.01$, and '*' for $p < 0.05$.

Random effects

Groups	*Name*	*Variance*	*Std.Dev.*
individual	(Intercept)	0.000	0.004

Number of obs: 43, groups: individual, 31

Conditional model

	Estimate	*SE*	*z-value*	*p-value*	
(Intercept)	68.000	11.181	6.082	<0.0001	****
Fish attack	-2.812	13.976	-0.201	0.8405	
Status empty	-52.200	15.412	-3.387	0.0007	***
Fish attack : Status	2.762	21.177	0.130	0.8962	

Supplementary Table 45. Generalized linear mixed effects model (GLMM) assessing significant differences in octopus web-over duration while hunting with extreme phenotypes or solitarily (hunting modes, 4 levels: OCTO, BTG, BG, and BTG&BG). An autocorrelation structure based on incremental 'blocks' was successfully fitted (Corr), field (Israel or Egypt) and scene (hunting group) were used as a nested random effect, in the formula: *web-over duration ~ hunting modes + ar1(block + 0|field/scene), family = Gamma(link = log)*. Presented are coefficient estimates, standard errors (SE), standard deviation (Std. Dev.), z-values, and p-values (Tukey-adjusted), with statistical significance shown as '****' for p < 0.0001, '***' for p < 0.001, '**' for p < 0.01, and '*' for p < 0.05.

Random effects

Groups	*Name*	*Variance*	*Std.Dev.*	*Corr*
scene:field	block3	0.061	0.246	0.46 (ar1)
field	block3	0.041	0.202	0.95 (ar1)

Number of obs: 476, groups: scene:field, 15; field, 2

Conditional model

	Estimate	*SE*	*z-value*	*p-value*	
(Intercept)	2.012	0.154	13.071	<0.0001	****
OCTO	-0.581	0.230	-2.524	0.0116	
BG	0.589	0.148	3.991	0.0001	***
BG&BTG	0.191	0.206	0.931	0.3521	

Tukey HSD Pairwise contrasts

	Estimate	*SE*	*z-value*	*p-value*	
BTG - OCTO	3.290	1.350	2.433	0.0710	
BTG - BG	-6.000	1.840	-3.269	0.0060	**
BTG - BTG&BG	-1.580	1.800	-0.877	0.8168	
OCTO - BG	-9.300	2.350	-3.958	0.0004	***
OCTO - BTG&BG	-4.870	2.070	-2.355	0.0860	
BG - BTG&BG	4.420	1.770	2.500	0.0599	

Supplementary Table 46. Generalized linear mixed effects model (GLMM) assessing significant differences in octopus web-over frequency while hunting with extreme phenotypes or solitarily (hunting modes, 4 levels: OCTO, BTG, BG, and BTG&BG). Field (Israel or Egypt), scene (hunting group), and block (subgroup), were used as a nested random effect, in the formula: *web-over frequency ~ hunting modes + (1|field/scene/block), family = nbinom1*. Presented are coefficient estimates, standard errors (SE), standard deviation (Std. Dev.), z-values, and p-values (Tukey-adjusted), with statistical significance shown as '****' for p < 0.0001, '***' for p < 0.001, '**' for p < 0.01, and '*' for p < 0.05.

Random effects

Groups	*Name*	*Variance*	*Std.Dev.*
block:scene:field	(Intercept)	0.000	0.000
scene:field	(Intercept)	0.041	0.203
field	(Intercept)	0.000	0.000

Number of obs: 114, groups: block:scene:field, 114; scene:field, 15; field, 2

Conditional model

	Estimate	*SE*	*z-value*	*p-value*	
(Intercept)	1.612	0.128	12.565	<0.0001	****
OCTO	0.367	0.200	1.841	0.0656	
BG	-0.662	0.191	-3.463	0.0005	***
BG&BTG	-0.179	0.218	-0.822	0.4112	

Tukey HSD Pairwise contrasts

	Estimate	*SE*	*z-value*	*p-value*	
BTG - OCTO	-2.225	1.267	-1.757	0.2945	
BTG - BG	2.425	0.729	3.325	0.0049	**
BTG - BTG&BG	0.822	0.984	0.835	0.8376	
OCTO - BG	4.650	1.146	4.058	0.0003	***
OCTO - BTG&BG	3.047	1.161	2.624	0.0431	*
BG - BTG&BG	-1.604	0.823	-1.949	0.2077	

Supplementary Table 47. Generalized linear mixed effects model (GLMM) and Tukey Honest Significant Differences (Tukey HSD) pairwise comparisons assessing significant differences in the rate of pulls between phenotypes. Rates of pulls were measured as the frequency of pulls within a finite time interval (200 seconds or 3.33 minutes) and the number of individuals present in that block, minus the puller ($\boldsymbol{n-1}$). Lastly, scene (hunting group), individual identity (individual), and block (hunting subgroup) were used as a nested random effect, with the formula: *pulling ~ phenotype + (1 | scene/individual/block) + offset(log((3.33) * (n - 1)), family = poisson*. Presented are coefficient estimates, standard errors (SE), standard deviation (Std. Dev.), z-values, and p-values (Tukey-adjusted), with statistical significance shown as '****' for p < 0.0001, '***' for p < 0.001, '**' for p < 0.01, and '*' for p < 0.05.

Random effects

Groups	***Name***	***Variance***	***Std.Dev.***
block:individual:scene	(Intercept)	0.2318	0.4815
individual:scene	(Intercept)	0.0022	0.0467
scene	(Intercept)	0.1290	0.3592

Number of obs: 289, groups: block:individual:scene, 289; individual:scene, 75; scene, 13

Conditional model

	Estimate	***SE***	***z-value***	***p-value***	
(Intercept)	-2.621	0.177	-14.807	<0.0001	****
barbel goatfish	0.681	0.173	3.927	0.0001	***
lyretail	0.001	0.269	0.004	0.9965	
octopus	0.209	0.195	1.072	0.2839	
blue goatfish	1.153	0.184	6.274	<0.0001	****
yellow goatfish	0.871	0.275	3.173	0.0015	**

Tukey HSD Pairwise comparisons

Contrast	***Estimate***	***SE***	***z-value***	***p-value***	
blacktip - barbel goatfish	-0.93698	0.26800	-3.49700	0.0063	**
blacktip - lyretail	-0.00114	0.25900	-0.00400	1.0000	
blacktip - octopus	-0.22360	0.21100	-1.06100	0.8970	
blacktip - blue goatfish	-2.08366	0.40100	-5.19400	<0.0001	****
blacktip - yellow goatfish	-1.33547	0.56200	-2.37500	0.1651	
barbel goatfish - lyretail	0.93585	0.33100	2.82600	0.0534	
barbel goatfish - octopus	0.71339	0.28200	2.52600	0.1165	
barbel goatfish - blue goatfish	-1.14667	0.40900	-2.80400	0.0568	
barbel goatfish - yellow goatfish	-0.39849	0.59100	-0.67400	0.9848	
lyretail - octopus	-0.22246	0.26800	-0.83100	0.9619	
lyretail - blue goatfish	-2.08252	0.40000	-5.20000	<0.0001	****
lyretail - yellow goatfish	-1.33433	0.58300	-2.29000	0.1982	
octopus - blue goatfish	-1.86006	0.38700	-4.80100	<0.0001	****
octopus - yellow goatfish	-1.11187	0.56400	-1.97000	0.3596	
blue goatfish - yellow goatfish	0.74819	0.59600	1.25500	0.8096	

Supplementary Table 48. Generalized linear mixed effects model (GLMM) and Tukey Honest Significant Differences (Tukey HSD) pairwise comparisons assessing significant differences in the rate of pulls between phenotypes. Rates of anchoring were measured as the frequency of anchors performed within a finite time interval (200 seconds or 3.33 minutes) and the number of individuals present in that block, minus the anchorer ($\boldsymbol{n-1}$). Lastly, scene (hunting group), individual identity (individual), and block (hunting subgroup) were used as a nested random effect, with the formula: *anchoring ~ phenotype + (1 | scene/individual/block) + offset(log((3.33) * (n - 1)), family = poisson*. Presented are coefficient estimates, standard errors (SE), standard deviation (Std. Dev.), z-values, and p-values (Tukey-adjusted), with statistical significance shown as '****' for p < 0.0001, '***' for p < 0.001, '**' for p < 0.01, and '*' for p < 0.05.

Random effects

Groups	***Name***	***Variance***	***Std.Dev.***
block:individual:scene	(Intercept)	0.000	0.000
individual:scene	(Intercept)	0.068	0.261
scene	(Intercept)	0.073	0.271

Number of obs: 289, groups: block:individual:scene, 289; individual:scene, 75; scene, 13

Conditional model

	Estimate	***SE***	***z-value***	***p-value***	
(Intercept)	-1.832	0.159	-11.495	<0.0001	****
barbel goatfish	-0.378	0.209	-1.808	0.0706	
lyretail	-0.120	0.278	-0.430	0.6675	
octopus	0.759	0.175	4.345	<0.0001	****
blue goatfish	-0.952	0.251	-3.791	0.0002	***
yellow goatfish	-0.921	0.405	-2.275	0.0229	*

Tukey HSD Pairwise comparisons

Contrast	***Estimate***	***SE***	***z-value***	***p-value***	
blacktip - barbel goatfish	0.666	0.364	1.832	0.4449	
blacktip - lyretail	0.238	0.541	0.441	0.9979	
blacktip - octopus	-2.402	0.625	-3.843	0.0017	**
blacktip - blue goatfish	1.299	0.355	3.660	0.0034	**
blacktip - yellow goatfish	1.274	0.442	2.883	0.0454	*
barbel goatfish - lyretail	-0.428	0.521	-0.821	0.9637	
barbel goatfish - octopus	-3.068	0.636	-4.821	<0.0001	****
barbel goatfish - blue goatfish	0.633	0.314	2.013	0.3346	
barbel goatfish - yellow goatfish	0.608	0.417	1.456	0.6922	
lyretail - octopus	-2.641	0.729	-3.621	0.0040	**
lyretail - blue goatfish	1.061	0.474	2.235	0.2215	
lyretail - yellow goatfish	1.035	0.558	1.856	0.4296	
octopus - blue goatfish	3.701	0.635	5.826	<0.0001	****
octopus - yellow goatfish	3.676	0.689	5.339	<0.0001	****
blue goatfish - yellow goatfish	-0.025	0.356	-0.071	1.0000	

Supplementary Table 49. Generalized linear mixed effects model (GLMM) and Tukey Honest Significant Differences (Tukey HSD) pairwise comparisons assessing significant differences in the rate of pulls between phenotypes. Rates of pulls were measured as the frequency of pulls within a finite time interval (300 seconds or 5 minutes) and the number of individuals present in that block, minus the puller ($\boldsymbol{n-1}$). Lastly, scene (hunting group), individual identity (individual), and block (hunting subgroup) were used as a nested random effect, with the formula: *pulling ~ phenotype + (1 | scene/individual/block) + offset(log((5) * (n - 1)), family = poisson*. Presented are coefficient estimates, standard errors (SE), standard deviation (Std. Dev.), z-values, and p-values (Tukey-adjusted), with statistical significance shown as '****' for p < 0.0001, '***' for p < 0.001, '**' for p < 0.01, and '*' for p < 0.05.

Random effects

Groups	*Name*	*Variance*	*Std.Dev.*
block:individual:scene	(Intercept)	0.121	0.347
individual:scene	(Intercept)	0.000	0.000
scene	(Intercept)	0.120	0.347

Number of obs: 218, groups: block:individual:scene, 218; individual:scene, 75; scene, 13

Conditional model

	Estimate	*SE*	*z-value*	*p-value*	
(Intercept)	-3.066	0.175	-17.518	<0.0001	****
barbel goatfish	0.620	0.168	3.681	0.0002	***
lyretail	-0.032	0.259	-0.125	0.9009	
octopus	0.167	0.187	0.892	0.3725	
blue goatfish	1.087	0.184	5.916	<0.0001	****
yellow goatfish	0.800	0.279	2.871	0.0041	**

Tukey HSD Pairwise comparisons

Contrast	*Estimate*	*SE*	*z-value*	*p-value*	
blacktip - barbel goatfish	-0.93698	0.26800	-3.49700	0.0142	**
blacktip - lyretail	-0.00114	0.25900	-0.00400	1.0000	
blacktip - octopus	-0.22360	0.21100	-1.06100	0.9502	
blacktip - blue goatfish	-2.08366	0.40100	-5.19400	<0.0001	****
blacktip - yellow goatfish	-1.33547	0.56200	-2.37500	0.2536	
barbel goatfish - lyretail	0.93585	0.33100	2.82600	0.0720	
barbel goatfish - octopus	0.71339	0.28200	2.52600	0.1586	
barbel goatfish - blue goatfish	-1.14667	0.40900	-2.80400	0.0996	
barbel goatfish - yellow goatfish	-0.39849	0.59100	-0.67400	0.9900	
lyretail - octopus	-0.22246	0.26800	-0.83100	0.9648	
lyretail - blue goatfish	-2.08252	0.40000	-5.20000	<0.0001	****
lyretail - yellow goatfish	-1.33433	0.58300	-2.29000	0.2624	
octopus - blue goatfish	-1.86006	0.38700	-4.80100	0.0001	***
octopus - yellow goatfish	-1.11187	0.56400	-1.97000	0.4417	
blue goatfish - yellow goatfish	0.74819	0.59600	1.25500	0.8248	

Supplementary Table 50. Generalized linear mixed effects model (GLMM) and Tukey Honest Significant Differences (Tukey HSD) pairwise comparisons assessing significant differences in the rate of pulls between phenotypes. Rates of anchoring were measured as the frequency of anchors performed within a finite time interval (300 seconds or 5 minutes) and the number of individuals present in that block, minus the anchorer ($\boldsymbol{n-1}$). Lastly, scene (hunting group), individual identity (individual), and block (hunting subgroup) were used as a nested random effect, with the formula: *anchoring ~ phenotype + (1 | scene/individual/block) + offset(log((5) * (n - 1)), family = poisson*. Presented are coefficient estimates, standard errors (SE), standard deviation (Std. Dev.), z-values, and p-values (Tukey-adjusted), with statistical significance shown as '****' for p < 0.0001, '***' for p < 0.001, '**' for p < 0.01, and '*' for p < 0.05.

Random effects

Groups	*Name*	*Variance*	*Std.Dev.*
block:individual:scene	(Intercept)	0.000	0.000
individual:scene	(Intercept)	0.063	0.251
scene	(Intercept)	0.063	0.251

Number of obs: 218, groups: block:individual:scene, 218; individual:scene, 75; scene, 13

Conditional model

	Estimate	*SE*	*z-value*	*p-value*	
(Intercept)	-2.257	0.158	-14.329	<0.0001	****
barbel goatfish	-0.372	0.212	-1.756	0.0790	
lyretail	-0.145	0.288	-0.503	0.6153	
octopus	0.748	0.176	4.258	<0.0001	****
blue goatfish	-0.906	0.251	-3.616	0.0003	***
yellow goatfish	-0.924	0.417	-2.215	0.0267	*

Tukey HSD Pairwise comparisons

Contrast	*Estimate*	*SE*	*z-value*	*p-value*	
blacktip - barbel goatfish	0.882	0.494	1.786	0.4745	
blacktip - lyretail	0.383	0.737	0.519	0.9954	
blacktip - octopus	-3.158	0.829	-3.808	0.0019	**
blacktip - blue goatfish	1.692	0.478	3.538	0.0054	**
blacktip - yellow goatfish	1.712	0.601	2.848	0.0502	
barbel goatfish - lyretail	-0.500	0.712	-0.702	0.9818	
barbel goatfish - octopus	-4.040	0.840	-4.808	<0.0001	****
barbel goatfish - blue goatfish	0.810	0.430	1.883	0.4129	
barbel goatfish - yellow goatfish	0.830	0.573	1.448	0.6976	
lyretail - octopus	-3.541	0.980	-3.612	0.0041	**
lyretail - blue goatfish	1.309	0.649	2.017	0.3326	
lyretail - yellow goatfish	1.329	0.766	1.736	0.5078	
octopus - blue goatfish	4.850	0.834	5.812	<0.0001	****
octopus - yellow goatfish	4.870	0.914	5.326	<0.0001	****
blue goatfish - yellow goatfish	0.020	0.496	0.040	1.0000	

Supplementary Table 51. Dyadic sample size in the dataset.

	blacktipgrouper	barbelgoatfish	lyretailgrouper	octopus	bluegoatfish	yellowgoatfish
blacktipgrouper	72	166	20	132	47	20
barbelgoatfish	166	27	28	95	47	8
lyretailgrouper	20	28	12	42	76	9
octopus	132	95	42	0	102	23
bluegoatfish	47	47	76	102	44	43
yellowgoatfish	20	8	9	23	43	4

Supplementary Table 52. Overview of the most important pull-anchor metrics, from the perspectives of the potential initiators (Pulling) and potential anchorers (Anchoring) of movement.

	Pulling		Anchoring	
	Definition	Interpretation	Definition	Interpretation
Frequency	p_i	Rate at which i successfully initiates movement in any individual j	a_i	Rate at which i successfully inhibits movement in any individual j
Initiation / Opportunity Frequency	$p\_i + an\_i$	Rate at which i initiates movement (successfully or unsuccessfully)	$a\_i + f\_i$	Rate of opportunities for i to inhibit movement in any individual j (successfully or unsuccessfully)
Efficiency	$p\_i / (p\_i + an\_i)$	Probability that when i initiates movement, any individual j will follow (probability of success)	$a\_i / (a\_i + f\_i)$	Probability that when any individual j initiates movement, it will be inhibited by i (probability of success)
General influence (leadership)	$p\_i + a\_i$	Rate at which i successfully initiates or inhibits movement in any individual j		

Supplemental Videos

Video S1. Example of a multispecific group hunting and typical movements, featuring all species categories analyzed in this study except yellow goatfish.

Video S2. Scene reconstruction example of the collective movement of multispecies groups.

Video S3. Examples of fish displacing other fish species.

Video S4. Examples of octopuses punching fish, thus displacing them.

Video S5. Example of a previous fish attack to structure that triggers a reaction from the octopus and consequent web-over over a food-baited structure.

Video S6. Typical movement dynamics and web-over temporal characteristics when octopuses are hunting alone.

Video S7. Typical movement dynamics and web-over temporal characteristics when octopuses are hunting in blue goatfish groups.

Data S1. Metadata file with group and subgroup (or blocks) details.

Data S2. Reconstructed 3D coordinates of all individuals in multispecific hunting groups.

Data S3. Displacements and punches annotations file.

Data S4. Web-over annotations file.

Data S5. Field experiment log file.
