# Supplementary material for: Multidimensional social influence drives leadership and composition-dependent success in octopus–fish hunting groups
Source: Nat Ecol Evol. 2024 Sep 23;8(11):2072–84. doi: 10.1038/s41559-024-02525-2 (PMC11541198; doi:10.1038/s41559-024-02525-2)
Supplement: Supplementary file 2 — Reporting Summary [file 41559_2024_2525_MOESM2_ESM.pdf]

nature portfolio

Corresponding author(s): Eduardo Sampaio

Last updated by author(s): Jun 14, 2024

Reporting Summary

Nature Portfolio wishes to improve the reproducibility of the work that we publish. This form provides structure for consistency and transparency in reporting. For further information on Nature Portfolio policies, see our Editorial Policies and the Editorial Policy Checklist.

Statistics

For all statistical analyses, confirm that the following items are present in the figure legend, table legend, main text, or Methods section.

n/a	Confirmed	
☐	☒	The exact sample size (*n*) for each experimental group/condition, given as a discrete number and unit of measurement
☐	☒	A statement on whether measurements were taken from distinct samples or whether the same sample was measured repeatedly
☐	☒	The statistical test(s) used AND whether they are one- or two-sided<br>*Only common tests should be described solely by name; describe more complex techniques in the Methods section.*
☐	☒	A description of all covariates tested
☐	☒	A description of any assumptions or corrections, such as tests of normality and adjustment for multiple comparisons
☐	☒	A full description of the statistical parameters including central tendency (e.g. means) or other basic estimates (e.g. regression coefficient) AND variation (e.g. standard deviation) or associated estimates of uncertainty (e.g. confidence intervals)
☐	☒	For null hypothesis testing, the test statistic (e.g. *F*, *t*, *r*) with confidence intervals, effect sizes, degrees of freedom and *P* value noted<br>*Give P values as exact values whenever suitable.*
☒	☐	For Bayesian analysis, information on the choice of priors and Markov chain Monte Carlo settings
☐	☒	For hierarchical and complex designs, identification of the appropriate level for tests and full reporting of outcomes
☐	☒	Estimates of effect sizes (e.g. Cohen's *d*, Pearson's *r*), indicating how they were calculated

Our web collection on statistics for biologists contains articles on many of the points above.

Software and code

Policy information about availability of computer code

Data collection	To transform 2D videos into 3D reconstructions, we used CVAT 2.3, colmap 3.9, and multiviewtracks 1.0 (https://github.com/pnuehrenberg/multiviewtracks)
Data analysis	We used Python 3.8, SciPy and R 4.3.1 to analyze the data. Code used is available at https://doi.org/10.6084/m9.figshare.26214830

For manuscripts utilizing custom algorithms or software that are central to the research but not yet described in published literature, software must be made available to editors and reviewers. We strongly encourage code deposition in a community repository (e.g. GitHub). See the Nature Portfolio guidelines for submitting code & software for further information.

Data

Policy information about availability of data

All manuscripts must include a data availability statement. This statement should provide the following information, where applicable:

- Accession codes, unique identifiers, or web links for publicly available datasets
- A description of any restrictions on data availability
- For clinical datasets or third party data, please ensure that the statement adheres to our policy

All data are available in Figshare, https://doi.org/10.6084/m9.figshare.26214830

Research involving human participants, their data, or biological material

Policy information about studies with human participants or human data. See also policy information about sex, gender (identity/presentation), and sexual orientation and race, ethnicity and racism.

Reporting on sex and gender	na
Reporting on race, ethnicity, or other socially relevant groupings	na
Population characteristics	na
Recruitment	na
Ethics oversight	na

Note that full information on the approval of the study protocol must also be provided in the manuscript.

Field-specific reporting

Please select the one below that is the best fit for your research. If you are not sure, read the appropriate sections before making your selection.

☐ Life sciences ☐ Behavioural & social sciences ☒ Ecological, evolutionary & environmental sciences

For a reference copy of the document with all sections, see nature.com/documents/nr-reporting-summary-flat.pdf

Ecological, evolutionary & environmental sciences study design

All studies must disclose on these points even when the disclosure is negative.

Study description	We recorded collective hunting events of octopuses and multiple fish species over ~120h of diving. We found and 3d reconstructed 13 unique groups that we split into blocks of 100 seconds (the length of the shortest hunting event), thus creating 107 subgroups nested within the first 13. We tested species and individual level differences in kinematics, as well as in dyadic dynamics of pull-anchor interactions (All details for species sample sizes and subgroup compositions are given in DataS1). We further looked at differences in kinematic and pull-anchor properties between groups depending on the presence or absence of the most extreme phenotypes (i.e. species). We then looked at the distribution of partner control mechanisms depending on species and evaluated individual-level behavior from the octopus. we investigated how the frequency and occurrence of partner control mechanisms by the octopus was affected by species presence and group kinematic variables such as displacement. Lastly, we looked at how web-over temporal characteristics changed in accordance to shifts in group composition, using the absence/presence of extreme phenotypes. We complemented these observations by performing a field experiment gauging how web-over temporal characteristics changed depending on the presence of food.
Research sample	Octopuses (Octopus cyanea) and multiple fish species, including : long barbel goatfish Parupeneus macronemus yellow and blue phase gold-saddle goatfish Parupeneus cyclostomus, lyretail grouper Variola louti, and blacktip grouper E. marginatus.
Sampling strategy	Using SCUBA, two divers surveyed coral reef areas for collective hunting activity. When found, given that these hunts are not stationary, we adopted a search-and-follow procedure while maintaining a distance of >5 m to minimize disturbing natural interactions. We used two full-frame Sony Alpha 7SII with Zeiss 2/25mm wide lenses mounted on an aluminum structure, as a stereocamera setup (hereafter 'Stereocamera Rig', Fig 1B). A third full-frame Sony Alpha 7SII with Sony f/4 24-70mm lens served as a focal camera for the octopus (hereafter 'Zoom Camera'). Sample size was inherently determined by the biological conditions, and species for which were present n < 5 were excluded from species-level analyses. The 120h of diving and changes in group composition provided a robust dataset to evaluate the different facets of collective hunting and individual behavior studied.
Data collection	Videos from the Stereocamera Rig were processed in our coding pipeline to create 3d reconstructions of individual tracks. Together with an additional ~30 minutes dataset in Egypt (El Quseir 26.1014° N, 34.2803° E), video from the Zoom Camera was used to quantify temporal characteristics of web-overs in solitary octopuses and punching variables.
Timing and spatial scale	Fieldwork spanned one month between 01-10-2018 and 01-11-2018 (29.5577° N, 34.9519° E, Eilat, Israel) in a total of ~120h of diving (~60h each diver). Dives were performed 2/3 dives/day, at relatively shallow depths (5-15m) allowing for ~2-3h underwater/day, complying with local scientific diving regulations. A field experiment was performed in Australia between 15-06-2023 and 15-07-2023.
Data exclusions	Data were not excluded from the analyses
Reproducibility	Analyses were rerun over several versions of R, producing similar results consistently.
Randomization	The original hunting groups filmed were divided into 100 second blocks of subgroups. We accounted for this dependence using auto-cirrelative structures to our statistical models whenever models were not outperformed for random effects modelling.

Blinding	Blinding was not possible due to the presence of other animals not being controlled, or possible to be occluded.

Did the study involve field work? ☐ Yes ☐ No

Field work, collection and transport

Field conditions	Coral reefs, water temperature between 24 and 29 degrees, visibility between 5 and 20 meters.
Location	Israel, Egypt, and Australia
Access & import/export	na
Disturbance	none

Reporting for specific materials, systems and methods

We require information from authors about some types of materials, experimental systems and methods used in many studies. Here, indicate whether each material, system or method listed is relevant to your study. If you are not sure if a list item applies to your research, read the appropriate section before selecting a response.

Materials & experimental systems

n/a	Involved in the study	
☒	☐	Antibodies
☒	☐	Eukaryotic cell lines
☒	☐	Palaeontology and archaeology
☐	☒	Animals and other organisms
☒	☐	Clinical data
☒	☐	Dual use research of concern
☒	☐	Plants

Methods

n/a	Involved in the study	
☒	☐	ChIP-seq
☒	☐	Flow cytometry
☒	☐	MRI-based neuroimaging

Animals and other research organisms

Policy information about studies involving animals; ARRIVE guidelines recommended for reporting animal research, and Sex and Gender in Research

Laboratory animals	No laboratory animals were used in this study
Wild animals	We observed collective hunts of octopuses (Octopus cyanea) and multiple fish species, including , long barbel goatfish Parupeneus macronemus yellow and blue phase gold-saddle goatfish Parupeneus cyclostomus, lyretail grouper Variola louti, and blacktip grouper E. marginatus. We estimate the age of the subjects between 6 months and 2 years. No direct experimental animal handling was performed. Structures with food and without food were presented to octopuses, following ethics permit from the authorities specified below.
Reporting on sex	Octopuses have little sexual dymorphism, so no information regarding sex was collected.
Field-collected samples	No field collected samples were used in this study.
Ethics oversight	Procedures were approved by the Max Planck Institute of Animal Behaviour, the Department of Agriculture and Fisheries Ethics Committee from Queensland, Australia, and the Great Barrier Reef Marine Parks Authority under permit G23/47925.1.

Note that full information on the approval of the study protocol must also be provided in the manuscript.

Plants

Seed stocks

Report on the source of all seed stocks or other plant material used. If applicable, state the seed stock centre and catalogue number. If plant specimens were collected from the field, describe the collection location, date and sampling procedures.

Novel plant genotypes

Describe the methods by which all novel plant genotypes were produced. This includes those generated by transgenic approaches, gene editing, chemical/radiation-based mutagenesis and hybridization. For transgenic lines, describe the transformation method, the number of independent lines analyzed and the generation upon which experiments were performed. For gene-edited lines, describe the editor used, the endogenous sequence targeted for editing, the targeting guide RNA sequence (if applicable) and how the editor was applied.

Authentication

Describe any authentication procedures for each seed stock used or novel genotype generated. Describe any experiments used to assess the effect of a mutation and, where applicable, how potential secondary effects (e.g. second site T-DNA insertions, mosiacism, off-target gene editing) were examined.
